# RIBEYE B-Domain Is Essential for RIBEYE A-Domain Stability and Assembly of Synaptic Ribbons

**DOI:** 10.3389/fnmol.2022.838311

**Published:** 2022-01-28

**Authors:** Soni Shankhwar, Karin Schwarz, Rashmi Katiyar, Martin Jung, Stephan Maxeiner, Thomas C. Südhof, Frank Schmitz

**Affiliations:** ^1^Institute of Anatomy and Cell Biology, Saarland University, Medical School, Homburg, Germany; ^2^Institute of Medical Biochemistry and Molecular Biology, Saarland University, Medical School, Homburg, Germany; ^3^Department of Molecular and Cellular Physiology, Stanford University School of Medicine, Stanford, CA, United States

**Keywords:** retina, inner ear, ribbon synapse, synaptic ribbon, RIBEYE A-domain, RIBEYE B-domain, CtBP2, presynaptic active zone

## Abstract

Synaptic ribbons are presynaptic specializations that define eponymous ribbon synapses. Synaptic ribbons are largely composed of RIBEYE, a protein containing an N-terminal A-domain and a carboxyterminal B-domain that is identical with CtBP2, a NAD(H)-binding transcriptional co-repressor. Previously we showed that synaptic ribbons are completely absent in RIBEYE knockout mice in which the RIBEYE A-domain-encoding exon had been deleted, but CtBP2 is still made, demonstrating that the A-domain is required for synaptic ribbon assembly. In the present study, we asked whether the RIBEYE B-domain also has an essential role in the assembly of synaptic ribbons. For this purpose, we made use of RIBEYE knockin mice in which the RIBEYE B-domain was replaced by a fluorescent protein domain, whereas the RIBEYE A-domain was retained unchanged. We found that replacing the RIBEYE B-domain with a fluorescent protein module destabilizes the resulting hybrid protein and causes a complete loss of synaptic ribbons. Our results thus demonstrate an essential role of the RIBEYE B-domain in enabling RIBEYE assembly into synaptic ribbons, reinforcing the notion that RIBEYE is the central organizer of synaptic ribbons.

## Introduction

Ribbon synapses are specialized chemical synapses found in the retina, inner ear, and pineal gland (Matthews and Fuchs, 2010; Moser et al., 2020). These synapses are continuously active and possess specialized active zones that allow fast, precisely timed signaling for prolonged periods of time. Exocytosis of synaptic vesicles occurs at the active zones close to the voltage-gated Ca^2+^-channels (Cav-channels) (Zenisek et al., 2000; Grabner and Moser, 2021). The active zones of ribbon synapses associate with large, electron-dense presynaptic specializations, the synaptic ribbons, that tether and deliver large numbers of synaptic vesicles to the active zone similar to an assembly line (Vaithianathan et al., 2019; Joselevitch and Zenisek, 2020). The ribbon-associated docked vesicles close to the active zone represent the vesicle pool that fuses with the fastest release kinetics (Moser et al., 2020; Grabner and Moser, 2021). Synaptic vesicles associated with more distant parts of the ribbon away from the active zone refill vacated release sites and promote continuous signaling (Zenisek et al., 2000; Vaithianathan et al., 2019; Joselevitch and Zenisek, 2020).

Synaptic ribbons are large presynaptic structures with a complex three-dimensional (3D) shape (Moser et al., 2020). The 3D shape and size of the ribbon are relevant for synaptic transmission/signaling because the ribbon geometry determines the pool size of ribbon-associated vesicles (Moser et al., 2020; Kesharwani et al., 2021). The photoreceptor synapse as the first synapse in the visual system transmits light stimuli to the inner retina. In the mouse retina, rod photoreceptors represent the major type of photoreceptors (95% of photoreceptors; Moser et al., 2020). Rod photoreceptor synapses are built in a fairly uniform manner. They contain a single, large active zone with a single large, horseshoe-shaped synaptic ribbon (Schmitz, 2009; Moser et al., 2020). Due to their large size and conspicuous horseshoe-shaped morphology, rod ribbons are particularly suitable for imaging analyses. In the inner retina, bipolar cell terminals possess multiple active zones with smaller synaptic ribbons that are morphologically and functionally more diverse than rod photoreceptor synapses in the outer retina (Moser et al., 2020).

Despite differences in size and shape, all ribbons contain the ribbon-specific protein RIBEYE (Schmitz et al., 2000; Moser et al., 2020), the core protein of all synaptic ribbons (Schmitz et al., 2000; Maxeiner et al., 2016; Becker et al., 2018; Jean et al., 2018). RIBEYE is evolutionarily conserved in vertebrates, and also serves as a major building block of synaptic ribbons in zebrafish (Lv et al., 2016; Sheets et al., 2017). Structurally, RIBEYE consists of a unique N-terminal, proline-rich A-domain, and a carboxyterminal B-domain that is identical (except for the N-terminal 20 amino acids) to the nuclear transcriptional co-repressor CtBP2 (Schmitz et al., 2000; Piatigorski, 2001). The RIBEYE B-domain/CtBP2 emerged from a family of D isomer-specific 2-hydroxyacid dehydrogenases (Goldberg et al., 1994). RIBEYE has multiple binding sites for other RIBEYE proteins both in the A- and B-domain of RIBEYE (Magupalli et al., 2008; Madison et al., 2013; Bellesis et al., 2018; Jecrois et al., 2021). However, the contribution of the RIBEYE A- and B-domains to the formation of synaptic ribbons remains unclear. Based on cell transfection experiments, the RIBEYE A-domain appears to serve mainly as a structural component of the synaptic ribbon scaffold (Schmitz et al., 2000; Magupalli et al., 2008), but it is unknown whether the RIBEYE A-domain alone is sufficient for the assembly of synaptic ribbons *in-situ* or whether this process also depends on RIBEYE B-domain.

In order to understand whether RIBEYE B-domain plays a role in the assembly of synaptic ribbons, we made use of RIBEYE^KI^ knockin (KI) mice (Maxeiner et al., 2016). In these KI mice, RIBEYE B-domain has been replaced by GCaMP3 in the KI allele, thus making these animals an ideal tool to study the role of the RIBEYE B-domain. We found that in the absence of the RIBEYE B-domain, synaptic ribbons are not assembled and the RIBEYE A-domain/GCaMP3 fusion protein is destabilized. Thus, the RIBEYE B-domain is essential for the assembly of synaptic ribbons.

## Materials and Methods

### Mice

All animal care and use procedures were reviewed and approved by the local animal authorities (Landesamt für Verbraucherschutz; Geschäftsbereich 3; 66115 Saarbrücken, Germany; GB 3-2.4.1.1-K110/180-07). Mice were anesthetized with isoflurane and killed by cervical dislocation in ambient light before organ collection. The RIBEYE knockin (KI) mice that were analyzed in the present study were generated by Maxeiner et al. (2016). In the RIBEYE KI, the alternative exon 1b of the mouse CtBP2/RIBEYE gene encoding RIBEYE A-domain was fused in frame with cDNA encoding for the Ca^2+^-indicator GCaMP3 (Tian et al., 2009) concluded by a STOP codon. As a consequence, the RIBEYE B-domain was replaced by GCaMP3 in the recombinant RIBEYE KI allele (Maxeiner et al., 2016). All possible genotypes at the recombinant RIBEYE locus (WT: RBE^WT/WT^; heterozygous KI: RBE^WT/KI^ and homozygous KI: RBE^KI/KI^) were analyzed in the RIBEYE KI mice as indicated in the respective experiments/figures. The genotypes were obtained by breeding heterozygous mice with each other (RBE^WT/KI^ X RBE^WT/KI^). Mice were kept under standard light/dark cycle and supported with standard food and water *ad libitum*.

### Methods

#### Embedding of Retinas and Immunocytochemistry on Semi-thin Retina Sections

Retina samples were processed for immunofluorescence microscopy (on resin sections) as previously described (Wahl et al., 2013, 2016; Dembla et al., 2014, 2018, 2020; Mukherjee et al., 2020). In brief, mice were anesthetized with isoflurane and killed by cervical dislocation. Eyes were enucleated within 5 min *post-mortem*. The anterior eyecup including the lens was removed by puncturing the isolated eye at the equatorial plane of the eye with a 20 Gauge needle and cutting along the equatorial plane with fine dissection scissors (FST, Heidelberg, Germany; No. 15024-10). The posterior eyecup with the attached retina was flash-frozen in liquid nitrogen-cooled isopentane. Lyophilization of the tissue was performed for ≈48 h with the tissue being continuously cooled by liquid nitrogen. Lyophilization of the samples was performed at a vacuum of ≈10^−7^ mbar using a TCP270 turbomolecular pump (Arthur-Pfeiffer-Vacuumtechnik, Wetzlar/Aßlar, Germany) controlled by a PKG020 Pirani-gold cathode gauge control unit and an oil diffusion pump (type DUO 004B; Arthur-Pfeiffer-Vacuumtechnik, Wetzlar/Aßlar, Germany). After lyophilization, samples were equilibrated to room temperature and infiltrated with Epon resin. For better infiltration with Epon, samples were equilibrated with Epon on a rotor (≈10 rpm) at 28°C for ≈24 h. Afterward, samples were degassed for 30 min in a vacuum chamber and were polymerized at 60°C for ≈24 h.

From the polymerized tissue blocks, 0.5 μm-thin (semi-thin) sections were cut with a Reichert ultramicrotome and collected on glass cover-slips, as previously described (Wahl et al., 2013, 2016). 0.5 μm-thin sections provide higher resolution compared to paraffin-embedded sections and cryosections (Punge et al., 2008). Epon resin was removed as described previously (Wahl et al., 2013, 2016; Dembla et al., 2018, 2020; Mukherjee et al., 2020). In brief, the Epon was removed from the 0.5 μm-thin resin sections by incubating the sections in the following solutions: sodium methanolate (30% solution in methanol; Sigma-Aldrich #8.18194) for 10 min; 1:1 mixture of xylol/methanol (10 min); acetone (2 × 10 min), tap water (1 min, 5×), PBS (1 min, 5×). For 3D SR-SIM analyses, 1.5 μm-thick sections instead of 0.5 μm-thin were used. The 1.5 μm-thick sections were treated for 12 min with sodium methanolate (instead of 10 min).

After resin removal, 0.5 μm-thin sections were incubated simultaneously with the indicated two primary antibodies that were generated in different animal species (mouse and rabbit) overnight at 4°C, as previously described (Wahl et al., 2013, 2016; Dembla et al., 2014, 2018). The next day, sections were washed several times with PBS to remove unbound primary antibodies and incubated with the corresponding fluorophore-conjugated secondary antibodies for 3 h at RT. After this incubation, sections were washed several times with PBS and mounted with an anti-fading solution, as previously described (Wahl et al., 2013, 2016; Dembla et al., 2018, 2020; Mukherjee et al., 2020). In control incubations, sections were incubated without primary antibody with the rest of the immunolabeling procedure remaining the same. Further controls were performed for the double immunolabeling experiments by setting individual laser power lines to zero with unchanged detection settings. These controls were done to make sure that the immunosignal in the channel of interest does not result from the neighboring detection channels (“bleed through controls”). Immunolabeled sections were analyzed by confocal microscopy, as described below.

#### Antibodies

##### Primary Antibodies

**Table:** 

Antibody	Source	Reference	Dilution
RIBEYE A-domain (6F4), mouse monoclonal IgG1 antibody(stock≈0.8 mg/ml)	Generated in the present study	Raised against a GST fusion protein encoding mouse RIBEYE(A): amino acids 83–211 (NP001164215)	1:100 (IF, WB) 1:500 (Cryo) 1:50 (EM)
RIBEYE(A), rabbit polyclonal (tau)	Lab-made	Maxeiner et al. (2016)	1:500 (IF)
RIBEYE(A)/ SySy, rabbit polyclonal	Synaptic Systems; Göttingen, Germany; 192103	Kerov et al. (2018)	1:500 (Licor)
RIBEYE B-domain (2D9), mouse monoclonal IgG2b antibody	Lab-made	Dembla et al. (2018)	1:200 (IF, Licor, WB)
Rhodopsin 1D4, mouse monoclonal	Gift: Dr. R.S. Molday	Hodges et al. (1988)	1:100 (IF)
PSD95, rabbit polyclonal (L667)	Dr. T.C. Südhof	Irie et al. (1997)	1:500 (IF)
Calbindin-D-28K (KD-15), rabbit polyclonal	Sigma; Darmstadt, Germany; C7354	Park et al. (2019)	1:100 (IF)
Glial Fibrillary Acidic Protein (GFAP), rabbit polyclonal	DaKo; Glostrup, Denmark; Z0334	Gruber et al. (2021)	1:500 (IF)
SV2, mouse monoclonal	Develop. Studies Hybridoma Bank; Univ. Iowa; Iowa City, IA, USA	Buckley and Kelly (1985)	1:20 (IF)
GFP, rabbit polyclonal	Abcam; Cambridge, UK; ab290	Yuan et al. (2021)	1:2,000 (Licor)
Actin, mouse monoclonal (clone C4)	Millipore; Molsheim, France (MAB1501)	Lessard (1988) and Mukherjee et al. (2020)	1:3,000 (WB, Licor)
PMCA2 ATPase, rabbit polyclonal	Thermo Fisher; Rockford, USA; PA1-915	Lin et al. (2021)	1:500 (IF)

##### Secondary Antibodies

**Table:** 

Antibody	Souce	Dilution
Donkey anti-mouse Alexa 568	Invitrogen; Karlsruhe, Germany; A-10037	1:1,000 (IF)
Donkey anti-mouse Alexa 488	Invitrogen; Karlsruhe, Germany; A-21202	1:1,000 (Cryo)
Chicken anti-rabbit Alexa 488	Invitrogen; Karlsruhe, Germany; A-21441	1:500 (IF)
Donkey anti-rabbit IRDye 800CW	LI-COR Biosciences; Bad Homburg, Germany; #92532213	1:5,000 (Licor)
Donkey anti-mouse IRDye 680LT	LI-COR Biosciences; Bad Homburg, Germany; #92568022	1:5,000 (Licor)
Goat anti-mouse peroxidase-conjugate (POX)	Sigma; Taufkirchen, Germany; A3673	1:3,000 (WB)
Goat anti-mouse conjugated to 10 nm colloidal gold	Sigma; Taufkirchen, Germany; G7652	1:100 (EM)

*Abbreviations: IF, Immunofluorescence on semi-thin resin sections; Cryo, Immunofluorescence on cryostat sections; Licor, Li-Cor Western blot; ECL, Western blot with enhanced chemiluminescence detection, EM, Electron microscopy. Miscellaneous reagents: Chameleon pre-stained protein ladder (Li-Cor Biosciences; Bad Homburg, Germany; #978-16526); Lectin PNA conjugated to Alexa 568 (Invitrogen; Karlsruhe, Germany; L32458)*.

#### Confocal Microscopy and Quantification of RIBEYE Immunofluorescence Signals and RIBEYE Puncta in the OPL

Confocal microscopy was performed with an A1R confocal microscope (Nikon), as previously described (Wahl et al., 2013, 2016; Eich et al., 2017; Dembla et al., 2018; Mukherjee et al., 2020). Image acquisition was performed in a blinded manner with the experimenter not knowing the identity (genotype) of the samples. Images were acquired with 60×/1.40 N.A. oil objective using laser excitation lines 488 nm and 561 nm under the control of the NIS Elements software (NIS Elements AR 3.2, 64 bit; Nikon). Identical conditions were maintained for confocal image acquisition using the “re-use” settings option of NIS Elements software for the comparative analyses of the different genotypes (RBE^WT/WT^, RBE^WT/KI^, and RBE^KI/KI^). For quantification of immunolabeled sections, NIH ImageJ software (“Fiji”) was used (Schindelin et al., 2012; Schneider et al., 2012).

For quantification of fluorescence intensities, the OPL was marked as the region of interest (ROI) by using the SV2- and PSD95 immunosignals as reference for the correct placement of the ROI. SV2 and PSD95 are well-characterized markers for photoreceptor synapses and produce a very typical staining pattern in the OPL (Buckley and Kelly, 1985; Koulen et al., 1998; Maxeiner et al., 2016). ROIs were managed with the Analyze-Tools-ROI Manager in NIH ImageJ. To analyze the fluorescence integrated density, a rectangular ROI was drawn around the entire OPL. The fluorescence intensity of the immunolabeled synaptic protein of OPL was determined as integrated density. The identical OPL-ROI was used for the analysis of immunolabeled sections of all genotypes. Quantifications were done blindly. Average integrated values were normalized. For this purpose, wild-type [RBE^WT/WT^] values were set to 100%, and heterozygous mice [RBE^WT/KI^], and homozygous knockin mice [RBE^KI/KI^] were related to wildtype values. Integrated density data were analyzed with Microsoft Excel. Individual datapoints were plotted as box and whisker plots in Origin Pro 2019b software. Statistical analyses for significance were performed as described below.

For counting the number of immunolabeled RIBEYE puncta per μm OPL, the length of OPL was derived from confocal NIS Elements software by using a known length distance for calibration. Confocal images were opened in ImageJ and the same OPL ROI was used for the integrated density measurements. After that, the image was duplicated (using the Image-Duplicate option of ImageJ) and puncta were counted automatically using the Process/Find Maxima-Point selection plugin (prominence 25, output/point selection including edge maxima), as described (Dembla et al., 2018). The average number of RIBEYE puncta was plotted as RIBEYE puncta per 100 μm length of OPL. Average puncta values for RBE^WT/WT^, RBE^WT/KI^, and RBE^KI/KI^ were plotted in Excel and data distribution was plotted in a box and whisker plot in Origin Pro 2019b software. Statistical analyses for significance were performed as described below.

#### Quantification of RIBEYE Immunosignals and RIBEYE Puncta Count in the IPL

For the analysis of fluorescence intensity of RIBEYE immunosignals in the IPL, integrated density measurements were performed on the acquired confocal images using Fiji NIH ImageJ (Schindelin et al., 2012; Schneider et al., 2012). The region of interest (ROI), i.e., the IPL, was selected with the help of the SV2- and PSD95 immunosignals (as described above for the OPL). A rectangular ROI was drawn around the SV2/PSD95 immunosignals in the IPL that served as a reference for the outline of the IPL. The size of the rectangular ROI was calibrated by using known distances that were measured by the confocal imaging and NIS Elements software. The same ROI was used for determining the RIBEYE puncta in the respective double-immunolabeled sections. The size of the IPL ROIs was kept identical for all genotypes. Integrated density was measured as described above for the OPL.

The average integrated values of RIBEYE A-domain and RIBEYE B-domain immunosignals in the IPL were normalized. The mean of the RBE^WT/WT^ values was set to 100% and RBE^WT/KI^, and RBE^KI/KI^ were compared with RBE^WT/WT^ wildtype values. Integrated density values were calculated in Microsoft Excel and individual data distribution was plotted as box and whisker plot with Origin Pro 2019b software. Statistical analysis was performed using Mann-Whitney *U*-test in Origin Pro 2019b or online Mann-Whitney *U*-test^1^ because data were non-normally distributed.

The number of immunolabeled RIBEYE A- and RIBEYE B-puncta in the IPL were counted automatically using the point selection plugin of ImageJ, as described above for the OPL. The puncta were counted in a rectangular area of 3,000 μm^2^ of an immunolabeled retina cross-section. Average RIBEYE puncta of RBE^WT/WT^, RBE^WT/KI^, and RBE^KI/KI^ retinas were plotted in Excel and individual data distribution was plotted in a box and whisker plot in Origin Pro 2019b software. Statistical analyses were done as described below.

#### 3D Super-Resolution Structured Illuminated Microscopy (3D SR-SIM) and Measurement and Quantification of Ribbon Contour Length

3D SR-SIM microscopy resolves immunolabeled objects beyond the diffraction limit of light (Schermelleh et al., 2010). For 3D SR-SIM microscopy, images were acquired from 1.5 μm-thick immunolabeled retina sections using the ELYRA PS1 setup (Carl Zeiss Microscopy GmbH), largely as previously described (Dembla et al., 2020; Mukherjee et al., 2020; Kesharwani et al., 2021). The 1.5 μm-thick sections, which were processed for 3D SR-SIM, were labeled only with a single primary antibody (anti-RIBEYE A-domain: 6F4 or anti-RIBEYE B-domain: 2D9, as described in the respective figures) and processed for indirect immunofluorescence microscopy as described above. Images were acquired with a 63×/1.4 NA oil (DIC) objective using the 561 nm laser line collected by an Andor iXon EM-CCD camera (100 ms exposure time), as previously described (Wahl et al., 2013, 2016; Dembla et al., 2020; Mukherjee et al., 2020; Kesharwani et al., 2021). For obtaining 3D SR-SIM images, *z*-stack images were taken at 125 nm intervals by using Zen 2012 software (black version). The entire thickness of the retinal section was scanned, and images were then processed for 3D SR-SIM. Single cropped anti-RIBEYE-immunolabeled synaptic ribbons were iteratively scanned to ensure that the entire immunolabeled synaptic ribbon was completely captured by the scans using ZEN 2.3 SP1 software (black version). From the *z*-stacks, the 3D views were created in transparent mode with ZEN 2.3 SP1 software. Maximum 2D projection images were generated from the 3D images of single, cropped synaptic ribbons, as previously described (Dembla et al., 2020; Mukherjee et al., 2020). The contour length of the maximum 2D projections was measured using the open polyline tool (Dembla et al., 2020; Mukherjee et al., 2020; Kesharwani et al., 2021). Average values were calculated and plotted in Microsoft Excel. Box and whisker plots were generated using Origin Pro 2019b software. Statistical analyses were performed as described below.

#### Immunolabeling of PNA-Stained Cone Synapses on Cryostat Sections of the Mouse Retina

Visualization of cone synapses with the fluorescent lectin PNA Alexa 568 and immunostaining of cone synapses was performed on ≈10 μm-thick cryostat sections, as previously described (Grabner et al., 2015). Cryostat sections were obtained from the retinas of RBE^WT/WT^ and RBE^KI/KI^ mice. For immunostaining/cone staining, cryo-sections were first heat fixed (10 min, 50°C) and then incubated with blocking buffer (0.5% BSA in PBS), for 1 h at RT. Next, sections were incubated with primary antibodies 6F4 (1:500 in blocking buffer, overnight, 4°C). Sections were washed three-four times with PBS and incubated simultaneously with donkey anti-mouse Alexa 488 secondary antibody (1:1,000 in blocking buffer) and PNA Alexa 568 (1:200 in blocking buffer) for 3 h at RT. After several washes with PBS, sections were mounted with anti-fading solution, as described above.

### Isolation of the Mouse Inner Ear and Whole-Mount Immunostaining of the Organ of Corti

For the dissection of the mouse cochlea, 4–8 weeks old RBE^WT/WT^ and RBE^KI/KI^ mice were used. Mice were anesthetized with isoflurane and killed by cervical dislocation/decapitation. After decapitation, mice heads were chilled for 15 min on ice. Isolation of the cochlea was performed, largely as described previously (Montgomery and Cox, 2016; Fang et al., 2019). In brief, first, the skull bone was removed after making a cut along the sagittal suture. The brain was removed and the cranial nerves were scrapped away from the temporal bone with an anatomical forceps (Schreiber Instrument, Fridingen Germany; SI Line; SI-14-1531). The petrous part of the temporal bone was transferred to a 2 ml microcentrifuge tube containing 4% PFA in PBS (pH 7.4) and incubated overnight at 4°C on a shaker. After several washes with PBS, the temporal bone was decalcified with a solution containing 120 mM ethylenediamine-tetraacetic acid (EDTA) in PBS, pH 7.4 (for 3 days, 4°C). EDTA solution was changed two times a day (after ≈8 h) (Montgomery and Cox, 2016). After decalcification, the temporal bone was washed again four-five times with PBS. The organ of Corti was isolated from the decalcified temporal bone as described previously (Montgomery and Cox, 2016; Fang et al., 2019). In brief, the vestibular portion of the petrous part of the temporal bone was held with forceps and cut away from the cochlea by a cut through the oval window and round window with a scalpel. After that, the bony capsule was removed and the cochlear turns were dissected. The isolated organs of Corti dissected from the basal, middle, and apical turn were placed in 24-well plates filled with PBS.

For immunostaining, samples were incubated with 300 μl blocking/permeabilization solution (0.5% BSA, 1% Triton X-100 in PBS; 1 h 30 min, RT, rotary shaker). Afterward, samples of the cochlea were double-immunolabeled with the indicated primary antibodies overnight at 4°C on a shaker. Antibodies were diluted in 0.5% BSA, 1%, Triton X-100 in PBS. After removing the unbound primary antibody with several washes in PBS, samples were incubated with corresponding secondary antibodies diluted in PBS containing 0.5% BSA and 1% Triton X-100 (3 h at RT on a shaker). After several washes with PBS, the immunolabeled cochleas were analyzed by confocal microscopy using a 60× water objective (Nikon NR Apo 60×/1.0W DIC N2). *Z*-stacks were acquired from the immunolabeled inner ear wholemounts and processed for maximum projection. Representative maximum projections are shown in Figure 13.

**Figure 1:**
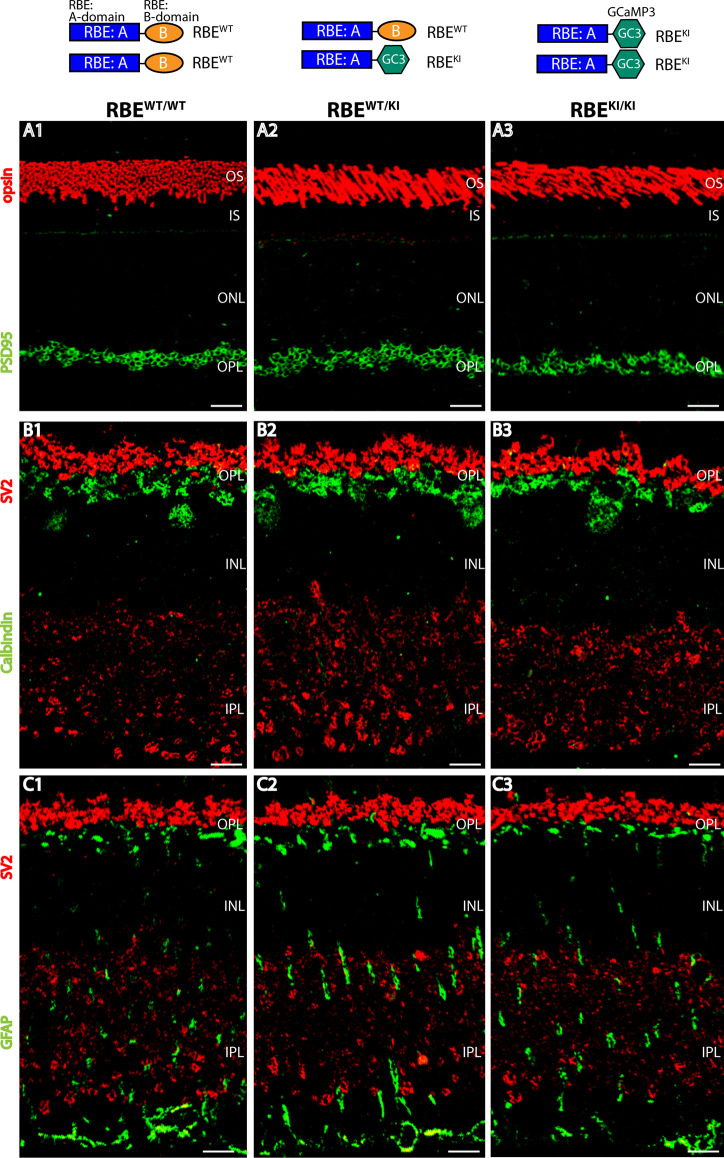
Normal organization of the retina in RIBEYE knockin (KI) mice. The top panel schematically depicts the analyzed genotypes (with the resulting RIBEYE proteins). **(A1–A3)** Semi-thin sections from the indicated littermate mice (RBE^WT/WT^, RBE^WT/KI^, and RBE^KI/KI^) were double-immunolabeled with anti-rhodopsin (red, to label photoreceptor outer segments) and anti-PSD95 antibody (green, to label photoreceptor synapses in the OPL. **(B1–B3)** Retinal semi-thin sections of the indicated genotypes double-immunolabeled with anti-SV2 (red, to label synaptic vesicles in OPL) and anti-calbindin (green, to label horizontal cells in the OPL). **(C1–C3)** Retinal semi-thin sections of the indicated genotypes double-immunolabeled with anti-SV2 (red, to label synaptic vesicles in OPL) and anti-GFAP (green, to label Müller glia cells). Abbreviations: OS, outer segment; IS, inner segment; ONL, outer nuclear layer; OPL, outer plexiform layer; INL, inner nuclear layer; IPL, inner plexiform layer; RBE, RIBEYE. Scale bar: 5 μm.

**Figure 2:**
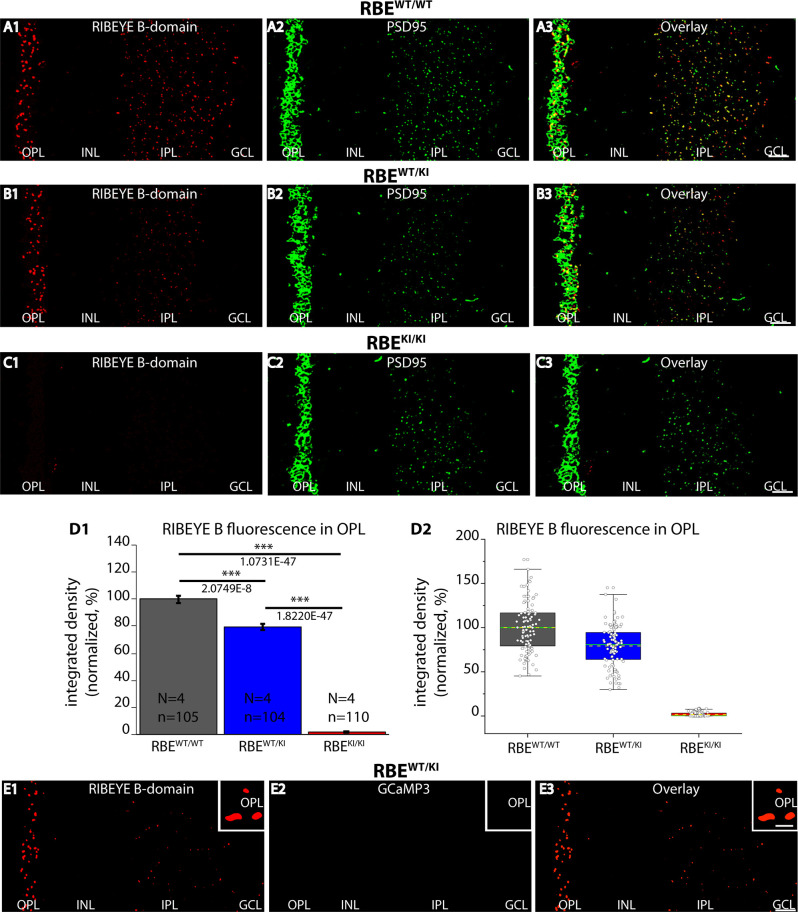
RIBEYE B-domain immunosignals are absent in photoreceptor synapses of RBE^KI/KI^ mice. **(A1–A3,B1–B3,C1–C3)** 0.5 μm-thin retina sections from the indicated littermate mice (RBE^WT/WT^, RBE^WT/KI^, and RBE^KI/KI^) were double-immunolabeled with mouse monoclonal anti RIBEYE B-domain (2D9) and rabbit polyclonal antibody against PSD95 (L667). **(C1)** RIBEYE B-domain immunosignals were completely absent in the OPL and IPL of RBE^KI/KI^ mice. **(D1)** Quantification of RIBEYE B-domain immunofluorescence signals in the OPL (as integrated density). Quantification confirmed the absence of RIBEYE B-domain immunosignals in the OPL of RBE^KI/KI^ mice. RIBEYE B-domain immunosignals in the OPL of RBE^WT/KI^ mice were reduced in comparison to RBE^WT/WT^ wild-type mice. Bar graphs show means ± S.E.M (***p ≤ 0.001). In the box and whisker plots **(D2)** of the data from **(D1)**, mean values are labeled by horizontal dotted yellow lines and median values by horizontal solid green lines. The boxes represent 25th-75th percentiles, and whiskers 1.5 times IQR. The statistical analysis was performed by Student’s *t*-test (for comparison of significance between RBE^WT/WT^ and RBE^WT/KI^ because data were normally distributed) and Kolmogorov-Smirnov test (for comparison between RBE^WT/KI^ and RBE^KI/KI^ and between RBE^WT/WT^ and RBE^KI/KI^ because RBE^KI/KI^ data were non-normally distributed). **(E1–E3)** 0.5 μm-thin retina sections from heterozygous RBE^WT/KI^ mice immunolabeled with anti-RIBEYE B-domain and visualized in the red channel **(E1)**. No GCaMP3 signal (direct fluorescence) is visible in the green channel in the immunolabeled semi-thin sections **(E2)**. Red and green channels were merged in **(E3)**. The insets show magnified regions of the OPL and confirm the absence of GCaMP3 signals at the ribbons in the OPL in semi-thin resin sections of the retina. Abbreviations: OPL, outer plexiform layer; INL, inner nuclear layer; IPL, inner plexiform layer; GCL, ganglion cell layer; RBE, RIBEYE; S.E.M., standard error of the mean; IQR, interquartile range; N = number of mice; n = number of analyzed confocal images. Scale bar: 5 μm.

**Figure 3:**
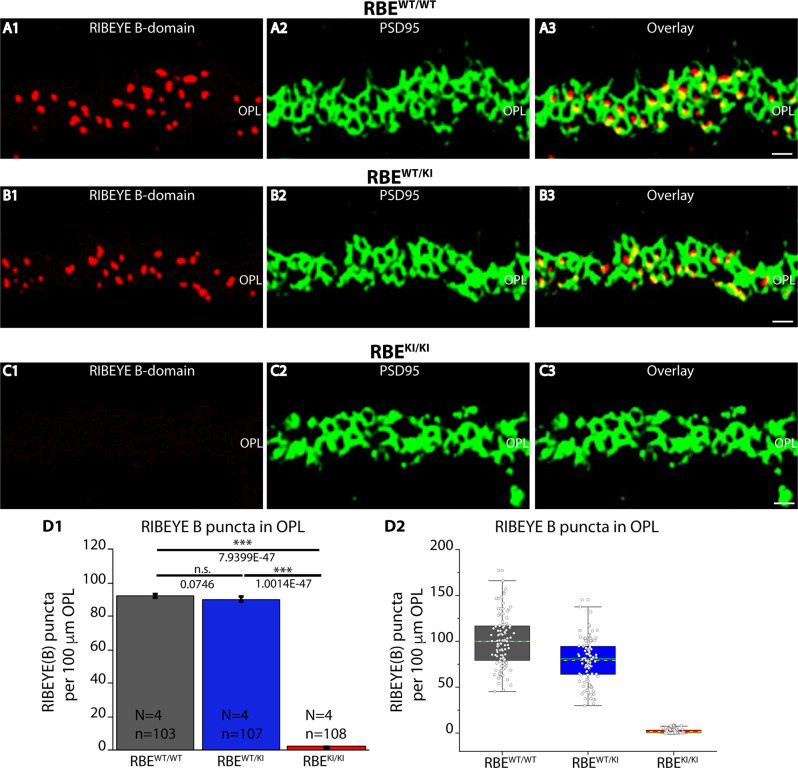
Quantification of RIBEYE B-domain puncta in the OPL of RBE^WT/WT^, RBE^WT/KI^, and RBE^KI/KI^ mice. **(A1–A3,B1–B3,C1–C3)** 0.5 μm-thin retina sections of RBE^WT/WT^, RBE^WT/KI^, and RBE^KI/KI^ mice were double-immunolabeled with mouse monoclonal antibody (2D9) against RIBEYE B-domain domain and rabbit polyclonal antibody against PSD95. The OPL was analyzed at high magnification to determine RIBEYE B-domain puncta number/density in the OPL. **(D1,D2)** Quantification of RIBEYE B-domain puncta in OPL in **(D1,D2)** demonstrates that the number of RIBEYE B-domain puncta is unchanged between RBE^WT/WT^ and RBE^WT/KI^. RIBEYE B-domain puncta were completely absent in the OPL of RBE^KI/KI^ mice. The bar graphs in **(D1)** show means ± S.E.M. (n.s., *p* > 0.05; ***p ≤ 0.001). In the box and whisker plots **(D2)** of the data from **(D1)**, means values are shown as horizontal yellow dotted line and median values by horizontal green solid line. The boxes represent the 25th-75th percentiles and whiskers represent 1.5 times IQR. The statistical analyses were performed by Mann-Whitney *U*-tests and Kolmogorov-Smirnov tests. Abbreviations: OPL, outer plexiform layer; RBE, RIBEYE; S.E.M., standard error of the mean; n.s. non-significant; IQR, interquartile range; *N* = number of mice; n = number of analyzed confocal images. Scale bar: 5 μm.

**Figure 4:**
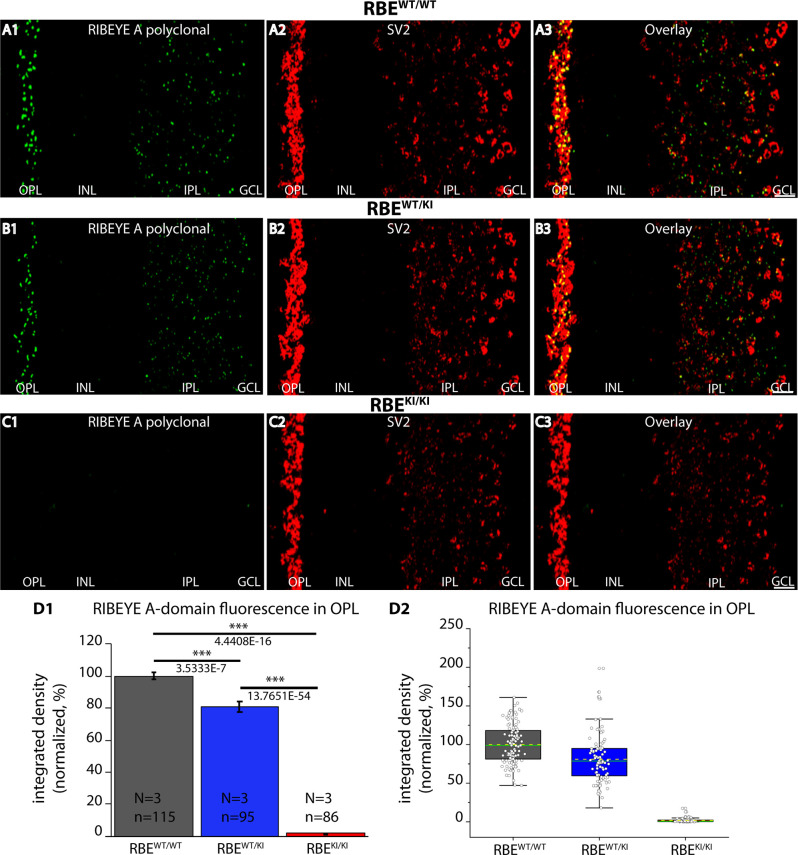
Absence of RIBEYE A-domain immunosignals in the retina of RBE^KI/KI^ mice. **(A1–A3,B1–B3,C1–C3)** 0.5 μm-thin retina sections from the indicated littermate mice (RBE^WT/WT^, RBE^WT/KI^, and RBE^KI/KI^) with mouse anti-SV2 and rabbit anti-RIBEYE A-domain (“tau”, Maxeiner et al., 2016). **(C1)** RIBEYE A-domain immunosignals were completely absent in the OPL and IPL of RBE^KI/KI^ mice. **(D1,D2)** Quantification of RIBEYE A-domain immunofluorescence signals (as integrated density). Quantification of the immunofluorescence signals in the OPL confirmed the absence of RIBEYE A-domain immunosignals in the OPL of RBE^KI/KI^ mice **(D1)**. RIBEYE A-domain immunosignals in the OPL of RBE^WT/KI^ mice were significantly reduced in comparison to RBE^WT/WT^ wild-type mice. Bar graphs in **(D1)** show means ± S.E.M. (***p ≤ 0.001). In the box and whisker plots **(D2)** of the data from **(D1)**, means values are shown as horizontal yellow dotted line and median values by horizontal green solid line. The boxes represent 25th-75th percentiles and whiskers represent 1.5 times IQR. The statistical analyses were performed with Mann-Whitney *U*-test and Kolmogorov-Smirnov tests because data were non-normally distributed. Abbreviations: OPL, outer plexiform layer; INL, inner nuclear layer; IPL, inner plexiform layer; GCL, ganglion cell layer; RBE, RIBEYE; S.E.M., standard error of the mean; IQR, interquartile range; N = number of mice; n = number of analyzed confocal images. Scale bar: 5 μm.

**Figure 5:**
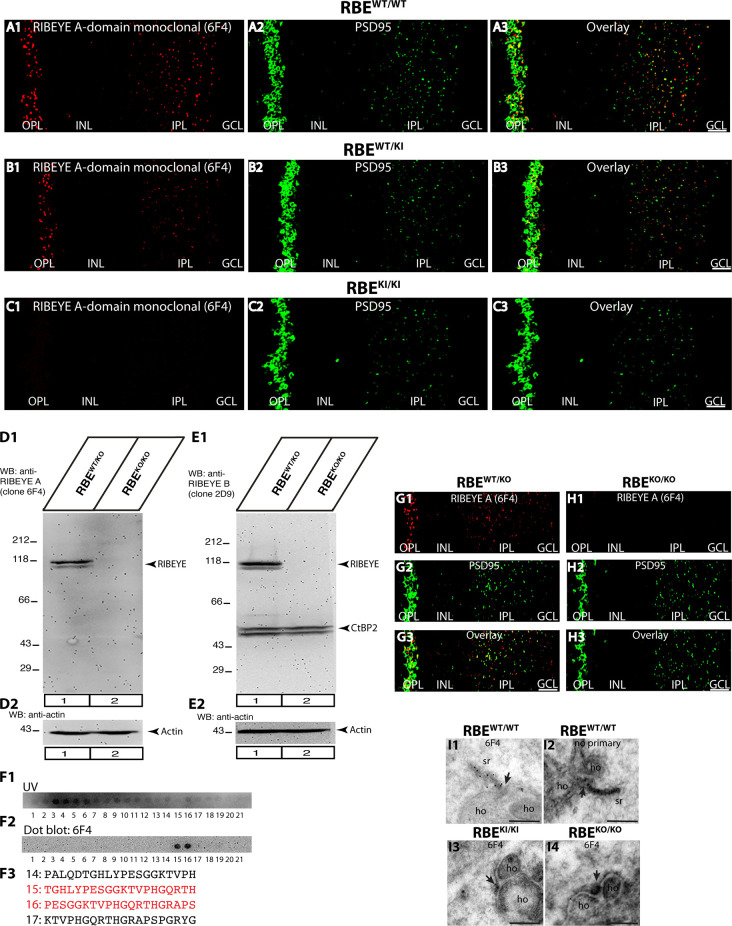
Confirmation of absence of RIBEYE A-domain immunosignals in the retina of RBE^KI/KI^ mice with a novel mouse monoclonal antibody against RIBEYE A-domain (6F4). **(A1–A3,B1–B3,C1–C3)** 0.5 μm-thin retina sections from the indicated littermate mice (RBE^WT/WT^, RBE^WT/KI^, and RBE^KI/KI^) with mouse anti-RIBEYE A-domain (clone 6F4) and rabbit anti-PSD95. **(C1)** RIBEYE A-domain immunosignals were completely absent in the OPL and IPL of RBE^KI/KI^ mice. The specificity of the novel anti-RIBEYE A-domain antibody 6F4 was verified by different experiments. **(D1,D2)** Retinal lysate from RBE^WT/KO^ and RBE^KO/KO^ mice were probed by WB analyses with anti-RIBEYE A-domain antibody 6F4 **(D1)** and anti-actin **(D2)**. Anti-actin signals were used as a loading control. **(E1,E2)** For comparison, retinal lysate from RBE^WT/KO^ and RBE^KO/KO^ mice were also probed by WB analyses with anti-RIBEYE B-domain antibody 2D9 **(E1)** and anti-actin **(E2)**, as previously described (Dembla et al., 2018). The anti-actin signals were used as loading controls. **(F1,F2)** Overlapping peptide spots, that cover the RIBEYE A-domain fusion protein against which the antibody was generated, were probed with RIBEYE A-domain 6F4 monoclonal antibody to map the precise binding site. **(F1)** Peptide spot array trans-illuminated with UV light to visualize the location of all peptide spots. **(F2)** Peptide spot array immunolabeled with anti-RIBEYE A-domain 6F4 antibody. The binding of the antibody was visualized by enhanced chemiluminescence. Peptide spots #15 and #16 were strongly immunoreactive with 6F4 antibodies. **(F3)** Peptide sequence of peptide spots #15 and #16 that strongly reacts with 6F4 antibody are highlighted in red in **(F3)**. **(G1–G3,H1–H3)** 0.5 μm-thin retina sections from RIBEYE knockout mice (RBE^KO/KO^) and control mice (RBE^WT/KO^) with mouse anti-RIBEYE A-domain (clone 6F4) and rabbit anti-PSD95. The 6F4 RIBEYE antibody generated a ribbon-typical immunostaining pattern only in the retina of the control mouse **(G1)** but not in the RIBEYE knockout mouse **(H1)** demonstrating the specificity of the 6F4 monoclonal antibody for RIBEYE. The PSD95 immunostaining is unchanged in both genotypes **(G2,H2,G3,H3)**. **(I1–I4)** Post-embedding immunogold labeling of rod photoreceptor synapses of the indicated genotype with anti-RIBEYE A-domain 6F4 monoclonal antibody. Arrowhead points to the active zone of photoreceptor ribbon synapses. Abbreviations: OPL, outer plexiform layer; INL, inner nuclear layer; IPL, inner plexiform layer; GCL, ganglion cell layer; sr, synaptic ribbon; ho, horizontal cells; RBE, RIBEYE. Scale bars: 5 μm (**A–C**; **G–H**); 300 nm **(I)**.

**Figure 6:**
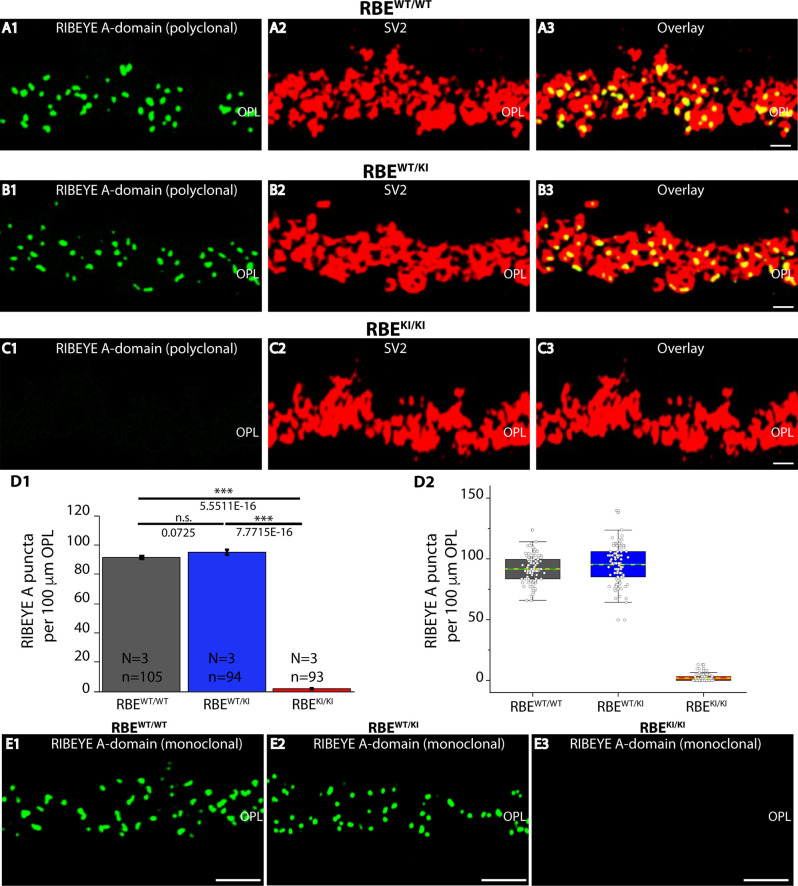
Quantification of RIBEYE A-domain puncta in the OPL of RBE^WT/WT^, RBE^WT/KI^, and RBE^KI/KI^ mice. **(A1–A3,B1–B3,C1–C3)** 0.5 μm-thin retina sections of RBE^WT/WT^, RBE^WT/KI^, RBE^KI/KI^ mice were double-immunolabeled with mouse monoclonal anti-SV2 and rabbit polyclonal antibody against RIBEYE A-domain (tau; Maxeiner et al., 2016). The OPL was analyzed at high magnification to determine RIBEYE A-domain puncta number/density in the OPL. **(D1,D2)** Quantification of RIBEYE A-domain puncta in OPL in **(D1,D2)** demonstrates that the number of RIBEYE A-domain puncta is unchanged between RBE^WT/WT^ and RBE^WT/KI^. RIBEYE B-domain puncta were completely absent in the OPL of RBE^KI/KI^ mice. The bar graphs in **(D1)** show means ± S.E.M (n.s., *p* > 0.05; ***p ≤ 0.001). In the box and whisker plots **(D2)** of the data from **(D1)**, means values are shown as horizontal yellow dotted line and median values by horizontal green solid line. The boxes represent the 25th-75th percentiles and whiskers represent 1.5 times IQR. The statistical analysis was performed by Student’s *t*-test for RBE^WT/WT^/RBE^WT/KI^ comparison because data were normally distributed. Kolmogorov-Smirnov tests were used for RBE^WT/WT^/RBE^KI/KI^ and RBE^WT/KI^ / RBE^KI/KI^ comparisons because RBE ^KI/KI^ data were non-normally distributed. **(E1–E3)** 0.5 μm-thin retina sections of RBE^WT/WT^, RBE^WT/KI^, and RBE^KI/KI^ mice were single-immunolabeled with mouse monoclonal antibody against RIBEYE A-domain clone 6F4. The two RIBEYE A-domain antibodies (monoclonal 6F4, polyclonal tau) produced very similar immunolabeling patterns also at the higher magnifications shown in Figure 6. Abbreviations: OPL, outer plexiform layer; RBE, RIBEYE; S.E.M., standard error of the mean; IQR, interquartile range; n.s., non significant; N = number of mice; n = number of analyzed confocal images. Scale bar: 5 μm.

**Figure 7:**
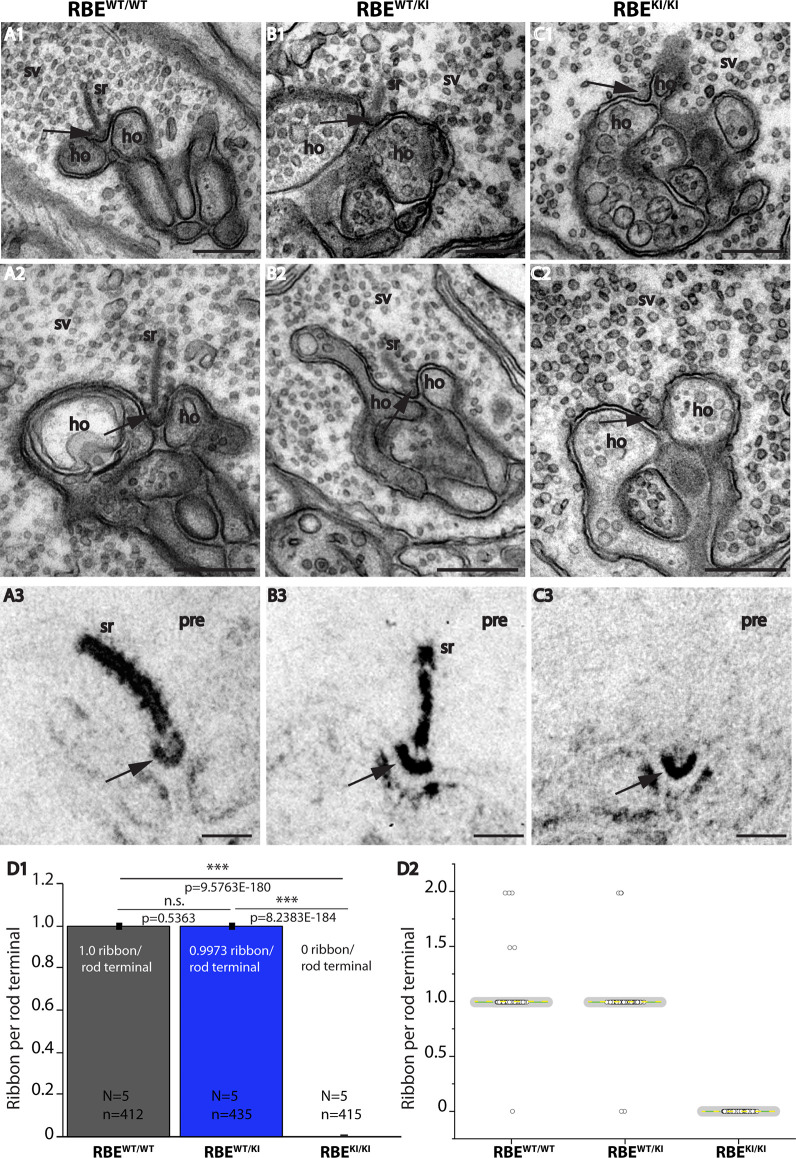
Absence of synaptic ribbons at the active zone of rod photoreceptor terminal from RBE^KI/KI^ mice as demonstrated by transmission electron microscopy. **(A–C)** Representative transmission EM (TEM) images of rod photoreceptor terminal RBE^WT/WT^, RBE^WT/KI^, and RBE^KI/KI^ mice. Panels **(A1,A2,B1,B2,C1,C2)** show conventional TEM images; panels **(A3,B3,C3)** show TEM images of E-PTA-stained samples. Arrows indicate the active zone of rod synapses. **(C1,C2,C3)** Synaptic ribbons are present at the active zone of RBE^WT/WT^ and RBE^WT/KI^ mice, but completely absent from the active zone of RBE^KI/KI^ mice. Except for the absence of synaptic ribbons, the ultrastructure of the rod photoreceptor synapses of RBE^KI/KI^ mice is comparable with control. **(D)** Quantification of the number of ribbons per rod terminal. Values in **(D1)** are means ± S.E.M. (n.s., *p* > 0.05; ***p ≤ 0.001). In the box and whisker plots **(D2)** of the data from **(D1)**, mean values are labeled by horizontal yellow dotted line and median values by horizontal green solid line. The box represents 25th-75th percentile, and whiskers represent 1.5 times of interquartile range. Statistical significance analyses were performed by Mann-Whitney *U*-tests and Kolmogorov-Smirnov tests. Abbreviations: sr, synaptic ribbon; ho, horizontal cells; SV, synaptic vesicles; pre, presynaptic; RBE, RIBEYE; S.E.M., standard error of the mean; N = number of mice, n = number of analyzed images; n.s., non-significant. Scale bar: 200 nm.

**Figure 8:**
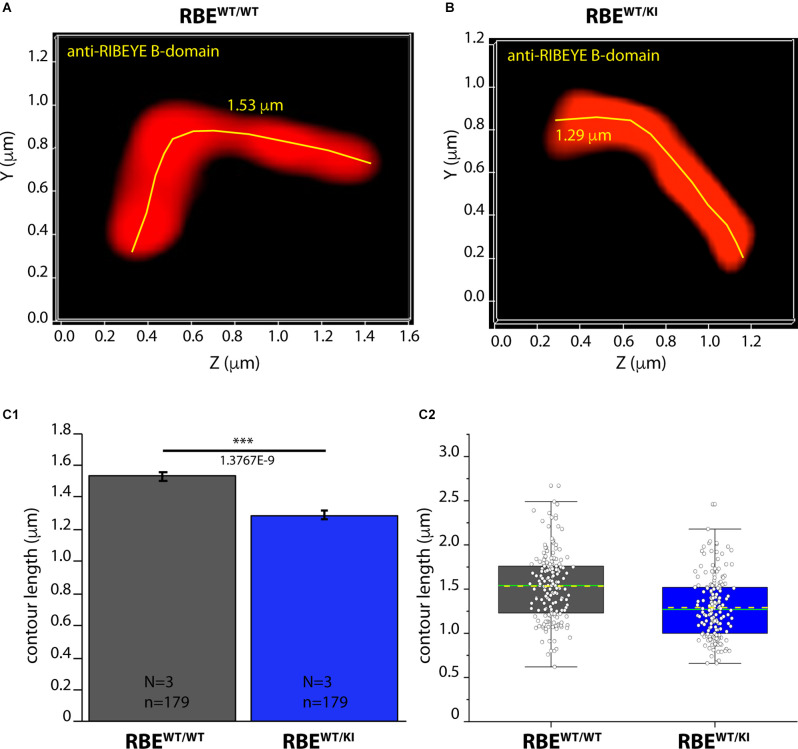
Contour length of synaptic ribbons is reduced in RBE^WT/KI^ mice in comparison to RBE^WT/WT^ mice [3D SR-SIM of ribbons immunolabeled with anti-RIBEYE(B)]. 1.5 μm-thin retina sections of mice from the indicated genotypes were immunostained with anti-RIBEYE B-domain antibody (2D9) and processed for 3D SR-SIM. **(A,B)** Representative 3D SR-SIM images of individual synaptic ribbons from rod photoreceptor synapses of RBE^WT/WT^ mice and RBE^WT/KI^ mice. The yellow lines specify examples of the contour length of rod synaptic ribbons. **(C1,C2)** Quantitative analysis of the contour lengths of rod photoreceptor synaptic ribbons were measured as maximum 2D projections from the 3D SR-SIM images, as previously described (Dembla et al., 2020; Mukherjee et al., 2020; Kesharwani et al., 2021). Values in **(C1)** are means ± S.E.M. (1.5336 ± 0.0281 μm in RBE^WT/WT^; 1.2908 ± 0.0258 μm in RBE^WT/KI^). In the box and whisker plot **(C2)** of the data from **(C1)**, mean values are indicated by dotted horizontal lines; median values by solid horizontal lines. Boxes represent the 25th-75th percentiles of data points and whiskers are equal to 1.5 times of the interquartile range (IQR). Mann-Whitney *U*-test was used to determine the statistical significance. Abbreviations: RBE, RIBEYE; S.E.M., standard error of the mean; N = number of mice; n = number of analyzed images; ***p ≤ 0.001.

**Figure 9:**
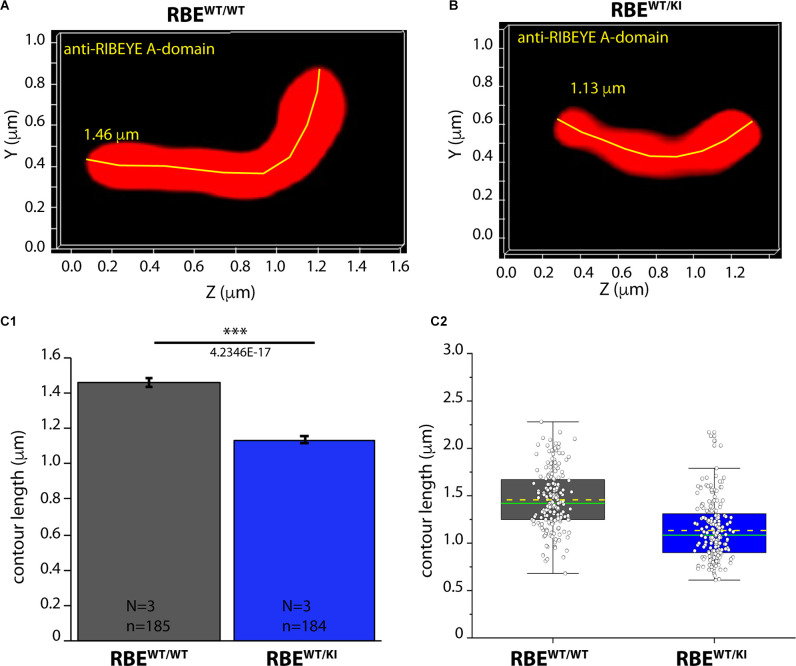
Contour length of synaptic ribbons is reduced in RBE^WT/KI^ mice in comparison to RBE^WT/WT^ mice [3D SR-SIM of ribbons immunolabeled with anti-RIBEYE(A)]. 1.5 μm-thin retina sections of mice from the indicated genotypes were immunostained with anti-RIBEYE A-domain mouse monoclonal antibody 6F4 and processed for 3D SR-SIM. **(A,B)** Representative 3D SR-SIM images of individual synaptic ribbons from rod photoreceptor synapses of RBE^WT/WT^ mice and RBE^WT/KI^ mice. The yellow line specifies examples of contour lengths of synaptic ribbons. **(C1,C2)** Quantitative analyses of the contour lengths of rod photoreceptor synaptic ribbons were measured as maximum 2D projections from the 3D SR-SIM images, as previously described (Dembla et al., 2020; Mukherjee et al., 2020; Kesharwani et al., 2021). Values in **(C1)** are means ± S.E.M. (1.4569 ± 0.0229 μm in RBE^WT/WT^; 1.1343 ± 0.0227 μm in RBE^WT/KI^). In the box and whisker plot **(C2)** of the data from **(C1)**, mean values are indicated by dotted horizontal lines; median values by solid horizontal lines. Boxes represent the 25th-75th percentiles of data points and whiskers are equal to 1.5 times of the interquartile range (IQR). Kolmogorov-Smirnov tests used to determine the statistical significance. Abbreviations: RBE, RIBEYE; S.E.M., standard error of the mean; N = number of mice; n = number of analyzed images; ***p ≤ 0.001.

**Figure 10:**
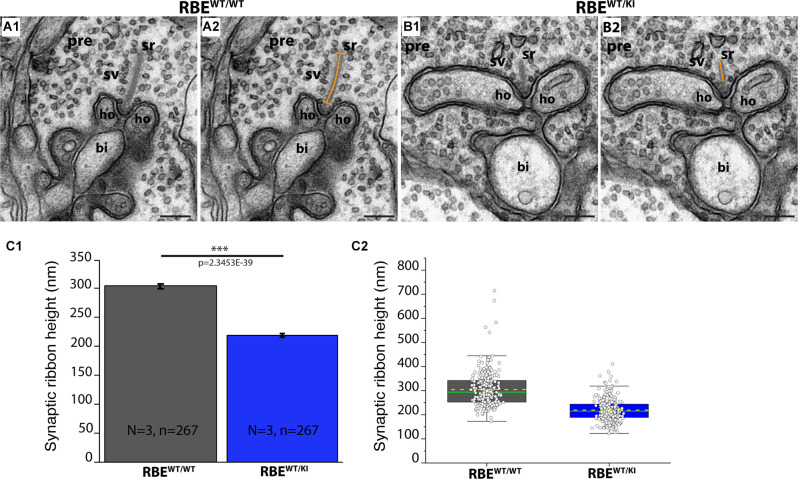
The height of rod photoreceptor synaptic ribbon is shorter in RBE^WT/KI^ mice in comparison to RBE^WT/WT^ mice. **(A,B)** Representative transmission EM images of cross-sectioned synaptic ribbon from rod-photoreceptor synapses of RBE^WT/WT^ mice **(A1,A2)** and RBE^WT/KI^ mice **(B1,B2)**. The dashed orange line in **(A2,B2)** demonstrates how synaptic ribbon height was determined. **(C)** Quantitative analyses. Values in **(C1)** are means ± S.E.M.: 304.6 nm ± 4.3 nm ribbon height in RBE^WT/WT^ mice; 219.1 nm ± 2.9 nm in RBE^WT/KI^ mice. In the box and whisker plot **(C2)** of the data from **(C1)**, mean values are indicated by dotted horizontal lines; median values by solid horizontal lines. Boxes represent the 25th-75th percentiles of data points and whiskers are equal to 1.5 times of the interquartile range (IQR). Kolmogorov-Smirnov tests were used to determine the statistical significance. Abbreviations: sr, synaptic ribbon; ho, horizontal cells; sv, synaptic vesicles; pre, presynaptic; bi, bipolar cell; RBE, RIBEYE; S.E.M., standard error of the mean; N = number of mice, n = number of analyzed images; ***p ≤ 0.001. Scale bar: 200 nm.

**Figure 11:**
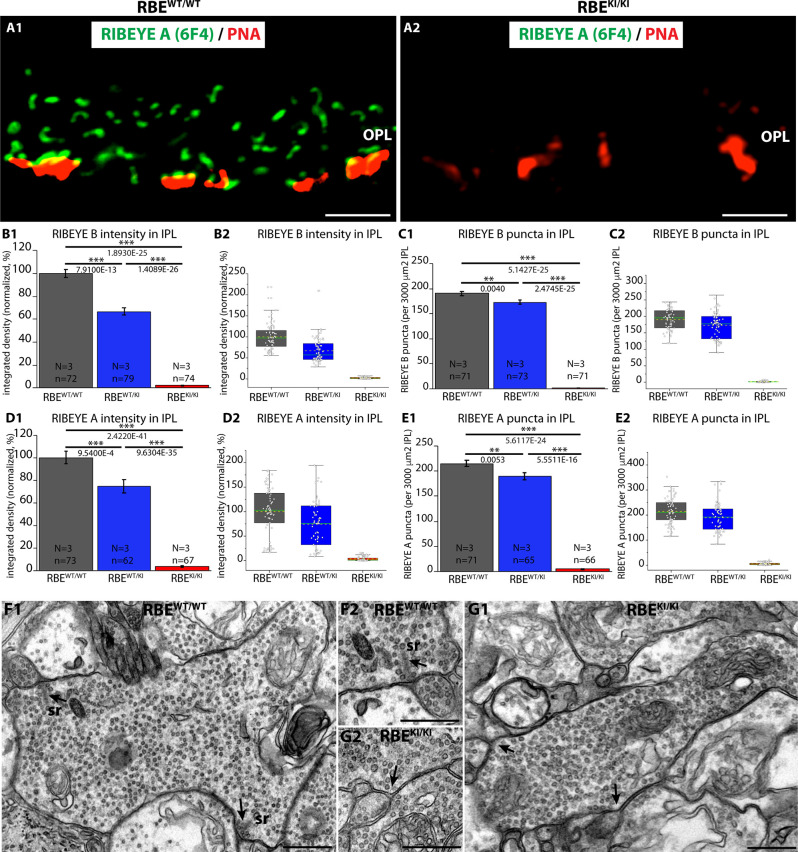
Absence of ribbons in cone synapses of RBE^KI/KI^ mice and quantification of RIBEYE immunosignals and RIBEYE puncta in the IPL of RBE^WT/WT^, RBE^WT/KI^, and RBE^KI/KI^ mice. **(A1,A2)** Cryostat sections of retinas obtained from the indicated genotypes were stained with PNA lectin Alexa 568 to visualize cone synapses in the OPL and immunolabeled with anti-RIBEYE 6F4 to visualize ribbons. Despite the clear presence of PNA-positive cone terminals, no ribbons were present in the OPL of RBE^KI/KI^ mice (Figure 11A). RBE^WT/WT^ mice served as controls (Figure 11A). **(B1,B2,C1,C2,D1,D2,E1,E2)** RIBEYE(B)/RIBEYE A-domain immunosignals and RIBEYE(B)/RIBEYE A-domain puncta in the inner plexiform layer that was double-immunolabeled with antibodies against RIBEYE(B)/PSD95 (Figures 2; Figures 3) and antibodies against RIBEYE(A)/SV2 (4; 6). **(B1,B2,D1,D2)** RIBEYE B-domain and RIBEYE A-domain immunosignals were completely absent in the IPL of RBE^KI/KI^ mice. In RBE^WT/KI^ mice, levels were inbetween the levels of RBE^WT/WT^ and RBE^KI/KI^ mice. **(C1,C2,E1,E2)** RIBEYE(B)/RIBEYE A-domain puncta were also completely absent in the IPL of RBE^KI/KI^ mice. In RBE^WT/KI^ mice, RIBEYE puncta (RIBEYE B-domain puncta and RIBEYE A-domain puncta) were between the levels of RBE^WT/WT^ and RBE^KI/KI^ mice. **(C1)** RIBEYE B-domain puncta in IPL: 190.1 ± 3.7 (mean ± S.E.M.) in RBE^WT/WT^, 172.9 ± 4.6 (mean ± S.E.M.) in RBE^WT/KI^ and 1.5 ± 0.2 (mean ± S.E.M.) in RBE^KI/KI^ mice. **(E1)** RIBEYE A-domain puncta in IPL: 215.0 ± 6.1 (mean ± S.E.M.) in RBE^WT/WT^, 189.5 ± 6.9 (mean ± S.E.M.) in RBE^WT/KI^ and 5.5 ± 0.6 (mean ± S.E.M.) in RBE^KI/KI^. Values in **(B1,C1,D1,E1)** are means ± S.E.M. In the box and whiskers plot **(B2,C2,D2,E2)** of the data from **(B1,C1,D1,E1)**, mean values are indicated by dotted horizontal lines; median values by solid horizontal lines. Boxes represent the 25th-75th percentiles of data points and whiskers are equal to 1.5 times of the interquartile range (IQR). To determine statistical significance, Mann-Whitney *U*-tests were performed for all comparisons of integrated densities. For RIBEYE B-domain puncta count, Student’s *t*-test was performed for RBE^WT/WT^ / RBE^WT/KI^ comparison because data were normally distributed; Mann-Whitney *U*-tests were performed for RBE^WT/WT^ / RBE^KI/KI^ and RBE^WT/KI^ / RBE^KI/KI^. For RIBEYE A-domain puncta count, Mann-Whitney *U*-tests were performed for RBE^WT/WT^/ RBE^WT/KI^, RBE^WT/WT^/RBE^KI/KI^ comparisons, Kolmogorov-Smirnov test for RBE^WT/KI^/ RBE^KI/KI^ because data were non-normally distributed. **(F,G)** Representative transmission EM images of cross-sectioned synaptic ribbons from rod bipolar synapses of RBE^WT/WT^ mice **(F1,F2)** and RBE^KI/KI^ mice **(G1,G2)**. Abbreviations: sr, synaptic ribbon; RBE, RIBEYE; S.E.M., standard error of the mean; N = number of mice, n = number of analyzed images; **p ≤ 0.01; ***p ≤ 0.001. The arrows in **(F1,F2,G1,G2)** point to the active zones. Scale bar: 500 nm.

**Figure 12:**
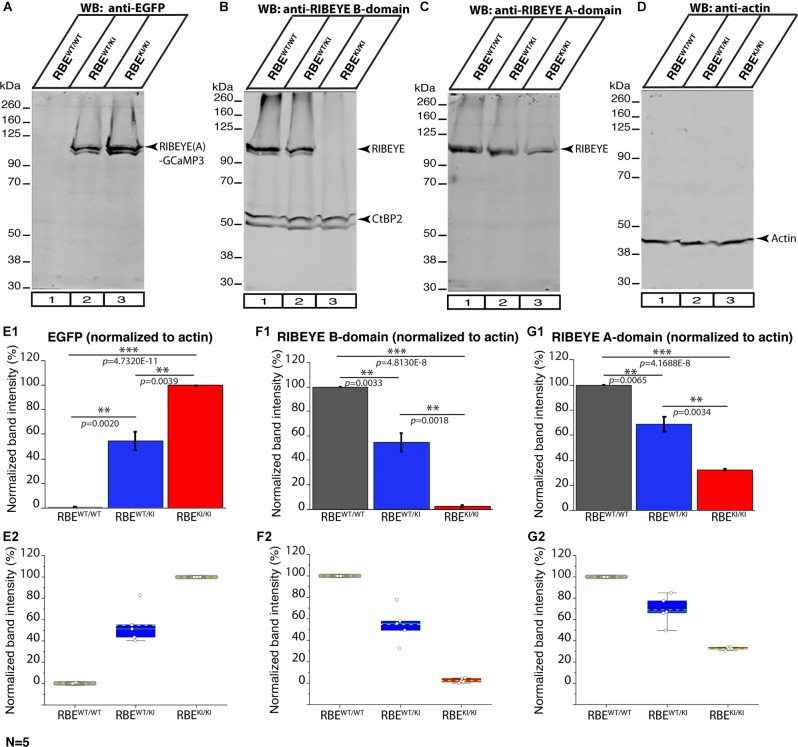
RIBEYE expression in the retina of RBE^WT/WT^, RBE^WT/KI^, and RBE^KI/KI^ mice (WB analyses; Li-Cor system). Retinal lysate from RBE^WT/WT^, RBE^WT/KI^, and RBE^KI/KI^ mice were probed by Western blot (WB) analyses with anti-GFP **(A)**, anti-RIBEYE B-domain **(B)**, anti-RIBEYE A-domain **(C)**, and anti-actin **(D)**. Anti-actin signals were used as loading control and for the normalization of GFP, RIBEYE B-domain, and RIBEYE A-domain WB bands. **(E1–G2)** Quantification of the indicated WB band intensities in RBE^WT/WT^, RBE^WT/KI^, and RBE^KI/KI^ mice using the Li-Cor system. The total fluorescent intensity values of the WB bands were computed using Image Studio Lite software and normalized to the loading control (actin). For the anti-GFP Western blots the RBE^KI/KI^ values, and for the anti-RIBEYE B-domain/RIBEYE A-domain Western blots the RBE^WT/WT^ values were set to 100% to better evaluate the relative changes in the different genotypes. Values in **(E1,F1,G1)** are means ± S.E.M. In the box and whisker plots **(E2,F2,G2)** of the data from **(E1,F1,G1)**, mean values are indicated by dotted horizontal lines; median values by solid horizontal lines. Boxes represent 25th-75th percentiles of data points; whiskers are equal to 1.5 times of the interquartile range (IQR). The expression of CtBP2 at ≈55 kDa did not differ significantly between the three analyzed genotypes RBE^WT/WT^ vs. RBE^WT/KI^: *p* = 0.9427; RBE^WT/KI^ vs. RBE^KI/KI^: *p* = 0.8108; RBE^WT/WT^ vs. RBE^KI/KI^: *p* = 0.6842. To determine the statistical significance, Student’s *t*-test was performed. Abbreviations: RBE, RIBEYE; S.E.M., standard error of the mean; N = number of the littermate mouse “triples” of the respective genotypes (RBE^WT/WT^, RBE^WT/KI^ and RBE^KI/KI^); **p ≤ 0.01; ***p ≤ 0.001.

**Figure 13:**
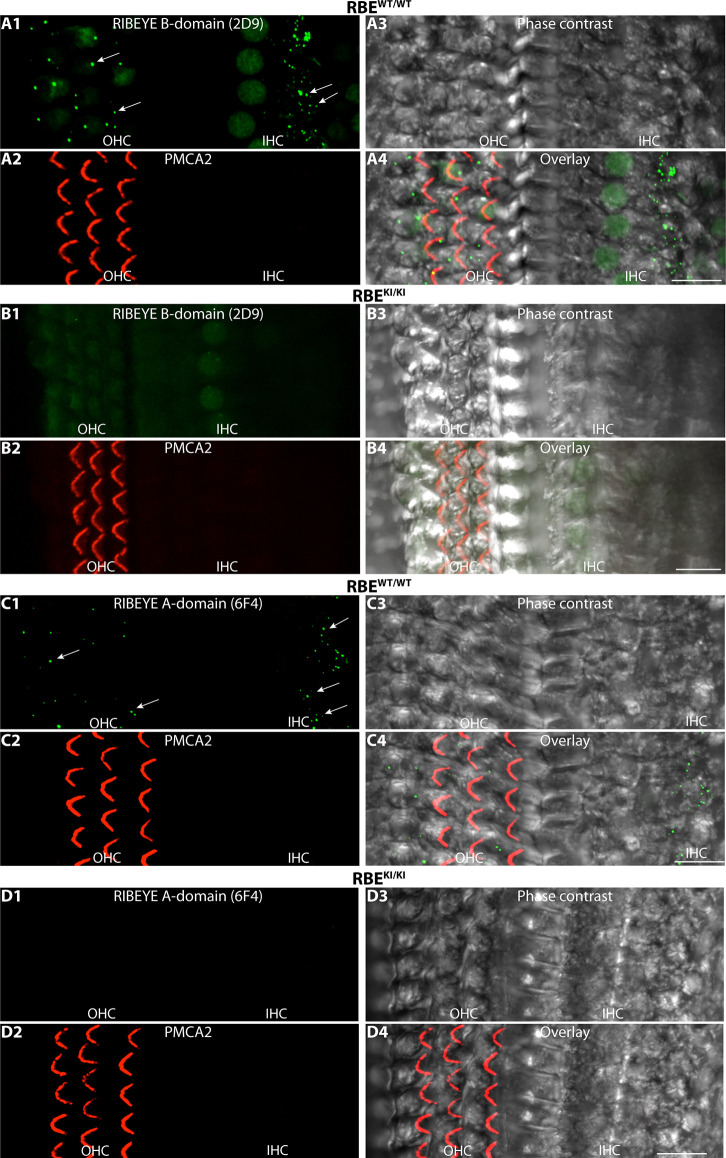
RIBEYE B-domain immunosignals are absent in inner and outer hair cells in the inner ear of RBE^KI/KI^ mice. **(A1–A4,B1–B4,C1–C4,D1–D4)** Whole mounts of mouse organs of Corti (isolated from RBE^WT/WT^ and RBE^KI/KI^ mice, as indicated) were double-immunolabeled with mouse monoclonal anti-RIBEYE B-domain antibody 2D9 **(A,B)**/mouse monoclonal anti-RIBEYE A-domain antibody 6F4 **(C,D)** and rabbit polyclonal antibody against PMCA2 to label stereocilia of outer hair cells (Watson and Temple, 2013; Bortolozzi and Mammano, 2018; Lin et al., 2021). The apical turns of the organs of Corti were used for the analyses. In the immunolabeled whole mounts, ribbons are clearly visible in the inner and outer hair cells (arrows) of RBE^WT/WT^ wild-type mice but not in RBE^KI/KI^ mice. All images are representative maximum projections obtained from the respective *z*-stacks. Abbreviations: IHC, inner hair cells; OHC, outer hair cells. Scale bar: 12 μm.

### Transmission Electron Microscopy (TEM)

#### Embedding of Retinas for Transmission Electron Microscopy

RBE^WT/WT^ wild-type, heterozygous RBE^WT/KI^, and homozygous RBE^KI/KI^ littermate mice were used for the comparative ultrastructural analyses of retinal synapses by transmission electron microscopy (TEM). Retina samples were processed and embedded for TEM, largely as previously described (Maxeiner et al., 2016). In brief, eyes were enucleated within 5 min *post-mortem*. Isolated eyes were hemisected coronally and the anterior eyecup, including the lens, was removed. The posterior eyecups with the attached retina were fixed with 4% (wt/vol) paraformaldehyde (in PBS, pH 7.4) and 2.5% (vol/vol) glutaraldehyde (in PBS) for 12 h each (at 4°C with constant gentle shaking). Retina samples were postfixed with 1% (wt/vol) OsO_4_, 1.5% (wt/vol) K_4_[Fe(CN)_6_] × 3H_2_O in 100 mM cacodylate buffer, pH 7.4 for 1 h (at 4°C on a rotating shaker). After three washes with 100 mM cacodylate buffer, pH 7.4, H_2_O and 50 mM Na-maleate in H_2_O (pH 5.0), samples were contrasted with 2% (wt/vol) uranyl acetate (UA) in 50 mM Na-maleate (pH 5.0) for 3 h (at 4°C on an orbital shaker). After several washes with maleate buffer and H_2_O, samples were dehydrated in an ascending, graded series of ethanol (30%, 50%, 70%, 80%, 90%, 99%) and pure acetone (15 min, each step at RT). Acetone was gradually replaced by a mixture of acetone and increasing volumes of Epon resin (3/1, 1/1, 1/3 (v/v); 1 h each, RT). Finally, samples were infiltrated with pure Epon resin overnight (RT, mild agitation). Epon-infiltrated samples were transferred to Silica embedding molds for polymerization. Polymerization was performed at ≈60°C for ≈24 h. Ultrathin sections (≈70 nm in thickness) were cut with a Reichert UCT ultramicrotome (Reichert-Jung). Sections were analyzed with a Tecnai Biotwin12 digital transmission electron microscope (FEI) equipped with a Megaview III digital camera (Gatan) and controlled by iTEM acquisition software (Olympus, Hamburg, Germany). The TEM microscope was operated at 100 kV.

Rod photoreceptor synapses in the outer plexiform layer (OPL) of the retina have a very typical ultrastructural appearance and can be clearly identified in the OPL by their typical appearance (Lagnado and Schmitz, 2015; Moser et al., 2020). The mouse retina is a rod-dominated retina. About 95% of photoreceptor synapses are made by rod photoreceptors. Rod photoreceptors possess a single large active zone that is typically occupied by a single large synaptic ribbon (Lagnado and Schmitz, 2015; Moser et al., 2020). Cone synapses are larger in size and possess multiple active zones with smaller synaptic ribbons (Lagnado and Schmitz, 2015; Moser et al., 2020). Ribbon synapses are also present in the inner plexiform layer (IPL) of the retina. Ribbons are present in most of the presynaptic terminals of retinal bipolar cells (Okawa et al., 2019). In general, synaptic ribbons in the IPL are smaller in size than in the OPL (Moser et al., 2020).

#### Staining of Retina Samples With Ethanolic Phosphotungstic Acid (E-PTA) for TEM

Ethanolic phosphotungstic acid (E-PTA) staining for TEM was performed as previously described (Bloom and Aghajanian, 1968; Limbach et al., 2011). For E-PTA electron microscopy, 4–5 months old littermate mice (RBE^WT/WT^, RBE^WT/KI^, and RBE^KI/KI^) were used for the analyses. The posterior eye-cups with the attached retina were immersed in 4% paraformaldehyde (PFA) in PBS (pH 7.4) (overnight at 4°C with mild shaking). After several washes with PBS, samples were dehydrated with an ascending ethanol concentration series (30%, 50%, 70%, 80%, 90%, 99%), 15 mins each step at RT. Next, samples were treated for 10 mins with absolute ethanol (100%) before samples were stained for 1.5 h in 1% phosphotungstic acid (w/v) in absolute ethanol that contained 5 drops of 95% absolute ethanol per 10 ml of staining solution (Bloom and Aghajanian, 1968; Limbach et al., 2011). Staining with E-PTA solution was performed for 1.5 h at RT on an orbital shaker. After removal of the E-PTA solution, retina samples were treated with ice-cold propylene oxide to avoid a strong exothermic reaction (Fry and Spira, 1980; Limbach et al., 2011). Propylene oxide was changed once and samples were subsequently incubated in propylene oxide for 30 min. Tissue was immersed in Epon solutions and embedded in Epon, as described above. Ultrathin sections were cut from the polymerized sample blocks and mounted on 100 mesh copper grids. Sections from E-PTA-stained samples were not further contrasted with UA or lead citrate and viewed with a Tecnai Biotwin12 (FEI) transmission electron microscope, as described above.

#### Quantification of the Presence of Synaptic Ribbons in Rod Photoreceptor Synapses and Determination of Ribbon Height Using TEM

The number of ribbons per rod terminal and ribbon height measurement in RBE^WT/WT^, RBE^WT/KI^, and RBE^KI/KI^ littermates were analyzed with TEM images from rod terminals acquired at a magnification of 43,000×. Only cross-sectioned rod terminals with a clearly visible active zone were included in the analyses. The average ratios of ribbon per rod terminal and ribbon height of RBE^WT/WT^, RBE^WT/KI^, and RBE^KI/KI^ mice were calculated and plotted in Microsoft Excel. For the analyses of ribbon number per rod terminal, RBE^WT/WT^ values were normalized to 1, and RBE^WT/KI^, RBE^KI/KI^ values were compared with RBE^WT/WT^. Box and whiskers were plotted in OriginPro 2019b software.

For measurement of synaptic ribbon height in rod photoreceptor synapses, only rod photoreceptor synapses of RBE^WT/WT^ and RBE^WT/KI^ mice were analyzed in which the active zone could be clearly visualized and in which the typical postsynaptic configuration consisting of horizontal and bipolar cells was visible (postsynaptic triad/tetrad). Ribbon height in the rod photoreceptor terminal was measured at a magnification of 43,000× by drawing a straight line from the base of the ribbon to the top of the ribbon using iTEM software. The length of the scale bar on the exported TEM images was used as a reference for length calibration.

#### Embedding of Retina Samples in LR Gold for Post-embedding Immunogold Electron Microscopy

Embedding of mouse retina and processing for immunogold labeling was performed as previously described (Schmitz et al., 2000; Wahl et al., 2013) with some modifications. Briefly, freshly isolated retinas were overnight fixed in 2% paraformaldehyde (in PBS, pH 7.4) at 4°C then 3× washed with PBS. Afterward, samples were dehydrated with increasing concentration of ethanol (30% ethanol 4°C, 10 min; pre-cooled 50%, 70%, 80% to 99% ethanol, 1 h at −20°C with mild agitation using a spinning wheel rotator). Samples were infiltrated with an increasing volume of LR gold (2/1, 1/2 (v/v); 1 h each, at −20°C) and then with pure LR gold overnight −20°C. After infiltrating samples with LR Gold that contained 0.1% benzil (w/v), samples were polymerized under UV light for 48 h at −20°C. After polymerization, ultrathin sections (≈70 nm in thickness) were cut with a Reichert-Jung ultramicrotome and collected on 100 mesh gold grids.

#### Post-embedding Immunogold Labeling

Post-embedding immunogold labeling was performed largely as previously described (Schmitz et al., 2000; Wahl et al., 2013). For immunolabeling, ultrathin sections of LR Gold-embedded tissue were first treated with 0.5% bovine serum albumin (BSA) in PBS, pH 7.4 (45 min, RT) to block nonspecific protein binding sites (Wahl et al., 2013). Then, sections were incubated with RIBEYE A-domain (6F4) primary antibody diluted 1:50 in blocking buffer (overnight at 4°C). After several washes with PBS, sections were incubated with goat anti-mouse secondary antibody conjugated to 10 nm gold particles (1:100 in blocking buffer, 1 h, RT). After several washes with PBS, immune complexes were fixed with 2.5% glutaraldehyde in PBS (15 min, RT). After removal of the PBS, sections were contrasted with 2% uranyl acetate (in H_2_O, 15 min, RT). As negative controls, primary antibodies were omitted with the rest of the immunogold labeling procedure remaining the same. Immunolabeled ultrathin sections were analyzed with a Tecnai Biotwin digital transmission electron microscope (FEI/ThermoFisher; Eindhoven, Netherlands), as described above.

#### Western Blot Analyses

Retinas from littermate mice of the indicated genotypes were isolated within 5 min *post-mortem* and dissolved in 200 μl Laemmli buffer. Protein lysates were solubilized by homogenization by up-/down pipetting in a 100 μl tip in Laemmli buffer and heated at 96°C for 10 min. The protein concentration of retina samples dissolved in the Laemmli buffer was determined with an amido black-based quantification method, as described (Dieckmann-Schuppert and Schnittler, 1997). Fifty microgram of retinal lysate was loaded per lane and separated by 10% acrylamide SDS PAGE. Proteins were electrotransferred to nitrocellulose membrane (Protran 0.45 μm) at 50 V for 5 h (4°C). For quantitative immunoblotting, the Li-Cor Western blot (WB) fluorescence detection system was used because of its wide dynamic range and high accuracy of quantitative WB measurements (Pillai-Kastoori et al., 2020). WB membranes were treated with 5% skimmed milk in PBS (blocking buffer) for 1 h at RT to block unspecific protein binding sites. The indicated primary antibodies were incubated overnight (at 4°C) at the indicated dilutions (in 3% skimmed milk in PBS). After several washes with PBS, WB membranes were probed with secondary antibody (donkey anti-rabbit IRDye 800CW, donkey anti-mouse IRDye 680 LT) diluted 1:5,000 in 3% skimmed milk in PBS (3 h, RT). After several washes, fluorescence signals were detected with Odyssey Infrared scanner and Odyssey software (Li-Cor Biosciences; Bad Homburg, Germany). The band intensities were quantified by using densitometry in Image Studio Lite software (Image Studio Lite 5.2 software; Li-Cor). The band density of the protein of interest was normalized to the actin signal density, that served as a loading control, in the same lane. For the quantitative analysis of RIBEYE A-domain and RIBEYE B-domain WB signals, RBE^WT/WT^ wild-type values were set to 100%, and RBE^WT/KI^ and RBE^KI/KI^ were compared to the respective wild-type values. For the analysis of GFP WB signals, RIBEYE^KI/KI^ values were set to 100%, and RBE^WT/KI^ and RBE^WT/WT^ values were related to this reference. The correlation coefficient of the results in the different experiments was calculated in Microsoft Excel. The correlation coefficient was high (*r* ≥ 0.8). Therefore, according to De Winter (2013), two-sample Student‘s *t*-test for non-equal variance was performed for determining the statistical significance. Normalized band signal graph was plotted in Microsoft Exel and statistical analysis was performed with OriginPro 2019b. In indicated experiments, Western blots were also processed for enhanced chemoluminescence (ECL) detection. In these cases, HRP-conjugated secondary antibodies were used (as summarized above). The respective ECL signals were scanned with a ChemiDoc^TM^ XRS Gel Doc system (Bio-Rad; Feldkirchen, Germany).

#### Peptide Arrays for Epitope Mapping

For antibody epitope mapping of RIBEYE A-domain monoclonal antibody 6F4, peptides of RIBEYE A-domain covering the N-terminus (amino acids 83–211 of mouse RIBEYE) with a length of 20 amino acids each and an overlap of five amino acids were synthesized on a hardened cellulose membrane with a ResPepSL-Synthesizer (Intavis Bioanalytical Instruments; Cologne, Germany), as described (Frank, 2002; Hilpert et al., 2007; Harsman et al., 2011). The membrane with the immobilized peptides was activated with methanol for 1 min at RT. After two brief washes with H_2_O, the membrane was equilibrated for 2 h with binding buffer (50 mM Tris-HCl, pH 7.5, 150 mM NaCl, 0.1% Triton X-100) with mild shaking at RT. Unspecific protein binding sites of the membrane were blocked by incubating membranes in a binding buffer containing 1 μM BSA (1 h, RT). Next, the membrane was incubated with primary antibody (6F4; 1:20,000 dilution in binding buffer, overnight, 4°C). Thereafter, the membrane was washed 3 × 10 min with binding buffer and incubated with HRP-conjugated goat anti-mouse antibody (1:10,000 in binding buffer) for 1 h at RT on a shaker. The bound antibody was visualized by enhanced chemoluminescence using a ChemiDoc^TM^ XRS Gel Doc apparatus (Bio-Rad, Feldkirchen, Germany). For the visualization of all peptide spots, the membrane was illuminated by UV light on the Bio-Rad GelDoc system.

#### Statistical Methods

Statistical analyses were performed using OriginPro 2019b software and GraphPad Prism 8.4.3. For all analyses, at least three independent experiments were performed for each experimental group, as indicated in the respective experiments. First, we tested whether the data from individual experiments could be pooled. For this purpose, data from the individual experiments of an experimental group were checked for normality test using the Shapiro-Wilk test. Depending on the results of the Shapiro-Wilk tests, data from the individual experiments were processed for different multiple comparison analyses. For normally distributed data, ANOVA with Bonferroni’s *post hoc* tests were performed; Kruskal-Wallis ANOVA test with Dunn’s *post-hoc* tests were performed for non-normally distributed data at a significance level of 0.05. When multiple comparisons within the same group did not differ significantly, the data within the same was pooled. If the data within the groups were significantly different, the mean values of the individual experiments were kept and used for comparison between groups. Then data were tested for significance test between two groups. When data were normally distributed, a two-sample Student‘s *t*-test (with equal/non-equal variance) was used. Variance was analyzed with a two sample test for variances (OriginPro) in order to select the appropriate Student’s *t*-test (equal or non-equal variances). For non-normally distributed data, nonparametric Mann-Whitney *U*-test and the two-sample Kolmogorov-Smirnov test were used, as indicated in the respective figures. Online Mann-Whitney *U*-test was performed at https://astatsa.com/WilcoxonTest/. Differences were considered to be statistically different with *p* < 0.05.

## Results

In the current study, we analyzed RIBEYE knockin (KI) mice, RBE^KI^ (Maxeiner et al., 2016) to study whether RIBEYE B-domain has a role in the assembly of synaptic ribbons. In the RIBEYE KI mice, RIBEYE B-domain has been replaced by the cDNA of GCaMP3 in the knockin (KI) allele (Maxeiner et al., 2016), making these KI mice an ideal model to study the function of the RIBEYE B-domain.

First, we checked whether the overall organization of the RBE^KI/KI^ retina is altered in comparison to littermate control mice**.** For this purpose, we double-immunostained retina sections obtained from mice with different RIBEYE genotypes (wildtype: RBE^WT/WT^; heterozygous knockin: RBE^WT/KI^ and homozygous knockin RBE^KI/KI^) with various antibodies, including antibodies against opsin, to label photoreceptor outer segments (Figure 1A), SV2 (Figures 1B,C) and PSD95 (Figure 1A) to label retinal synapses, GFAP (Figure 1C) to label retinal Müller glia cells, and calbindin (Figure 1B) to mark horizontal cells. Using these markers, we did not observe gross alterations/differences in the retinal organization in mice with different RIBEYE genotypes (RBE^WT/WT^; RBE^WT/KI^, and RBE^KI/KI^), arguing that the RBE^KI^ allele does not grossly affect the retinal organization. Similarly, the retinas of RIBEYE knockout mice, in which the RIBEYE protein was completely deleted, did not show gross alterations in retinal morphology and organization (Maxeiner et al., 2016).

Next, we checked for the expression of the RIBEYE B-domain in the RBE^KI^ mice (Figure 2) using a well-characterized mouse monoclonal antibody (clone 2D9) against the RIBEYE B-domain (Dembla et al., 2018). Both in wild-type RBE^WT/WT^ mice and in heterozygous RBE^WT/KI^ littermate mice, we observed strong punctate, ribbon-typical RIBEYE B-domain immunosignals in both synaptic layers (OPL and IPL) of the retina (Figure 2). The RIBEYE B-domain immunosignal was completely absent in the OPL and IPL of homozygous RBE^KI/KI^ mice (Figure 2C1). The absence of RIBEYE B-domain immunosignals in the homozygous RBE^KI/KI^ was expected because the RIBEYE B-domain was replaced in the RIBEYE KI allele by GCaMP3. Particularly strong RIBEYE B-domain immunosignals were observed in the OPL (Figure 2) of wildtype RBE^WT/WT^ mice and heterozygous RBE^WT/KI^ littermate mice because synaptic ribbons in rod synapses of the OPL are particularly large (Moser et al., 2020). For quantitative analyses, we thus first focused on the OPL of the retina in which the photoreceptor ribbon synapses are located.

Quantification of immunosignals in the OPL revealed that the RIBEYE B-domain immunosignals in the OPL were weaker in heterozygous RBE^WT/KI^ mice compared to littermate wildtype RBE^WT/WT^, and confirmed the complete absence of RIBEYE B-domain immuno-signals in RBE^KI/KI^ mice (Figure 2D). Of note, the GCaMP3 moiety in the RIBEYE KI allele does not generate any fluorescence signals in resin sections of the immunolabeled retinas as shown by control incubations (Figure 2E).

The number and density of RIBEYE B-domain positive puncta in the OPL were determined on RIBEYE B-domain immunolabeled sections at higher magnifications that allow clear identification of single puncta (Figures 3A–C). Quantitative analyses revealed that the number of RIBEYE B-domain-positive puncta in the OPL were indistinguishable between RBE^WT/WT^ and heterozygous RBE^WT/KI^ mice (Figure 3D), but were completely absent from the OPL of homozygous RBE^KI/KI^ mice as expected (Figure 3D).

The RIBEYE B-domain antibody 2D9 also detects CtBP2 that is ubiquitously expressed as a nuclear co-repressor (Dembla et al., 2018). In the 0.5 μm thin retina sections, we did not observe a nuclear staining, similar to other studies (Dembla et al., 2018; Kesharwani et al., 2021), most likely because the focal concentration of CtBP2 in the nucleus is too low.

Is the RIBEYE A-domain still assembled into ribbon-like structures in RBE^KI^ mice? To address this question, we stained retinal sections with a polyclonal antibody against RIBEYE A-domain (Maxeiner et al., 2016). As mentioned above, the RIBEYE A-domain is still present on the RBE KI allele, in contrast to the RIBEYE B-domain that has been replaced. In wildtype RBE^WT/WT^ mice and heterozygous RBE^WT/KI^ littermate mice, we observed a strong punctate ribbon-typical RIBEYE A-domain immunosignal in both synaptic layers of the retina (OPL and IPL) (Figures 4A–C). Surprisingly, the RIBEYE A-domain signal was completely absent in the OPL of RBE^KI/KI^ mice (Figures 4C,D). Immunolabeling with an anti-SV2 antibody served as a control (Figure 4), demonstrating that synapses were still present as the SV2 immunosignals were unchanged in all genotypes (Figure 4).

The results obtained with the rabbit polyclonal antibody against the RIBEYE A-domain (Figures 4A–C) were confirmed with a newly generated mouse monoclonal RIBEYE A-domain antibody, clone 6F4 (Figure 5). The immunostaining pattern obtained with this antibody was indistinguishable from that produced with the polyclonal RIBEYE A-domain antibody, validating the absence of RIBEYE A-domain-positive puncta in the OPL and IPL of RBE^KI/KI^ mice (Figure 5C). In contrast, the RIBEYE A-domain immunostaining in the OPL and IPL of heterozygous RBE^WT/KI^ and wildtype RBE^WT/WT^ mice appeared unchanged. The newly generated mouse monoclonal RIBEYE A-domain antibody 6F4 is directed against a peptide stretch in the A-domain of RIBEYE (amino acid 83–amino acid 211 of mouse RIBEYE). The precise binding site of the antibody was determined by overlapping peptide dot blot experiments (Figure 5F). The specificity of the 6F4 antibody for RIBEYE was further demonstrated by Western blotting and immunofluorescence experiments on RIBEYE knockout mouse retina samples (Figures 5D,G,H). For the Western blotting experiments, the RIBEYE B-domain 2D9 monoclonal antibody was used for comparison. Both antibodies detected the full-length RIBEYE band at ≈120 kDa in control retinas, but not in retinas obtained from RIBEYE knockout mice (Figures 5D1,E1). The RIBEYE B-domain antibody 2D9 also detected CtBP2 at ≈50kDa in control and RIBEYE knockout retinas (Figure 5E1). CtBP2 is largely unaffected by the RIBEYE deletion in the RIBEYE knockout mice, as previously demonstrated (Dembla et al., 2018). The specificity of the RIBEYE A-domain antibody 6F4 was further confirmed by post-embedding immunogold labeling (Figure 5I). 6F4 antibody labeled synaptic ribbons in the control retina (Figure 5I1), but not in photoreceptor synapses of RIBEYE knockout retinas (Figure 5I4).

RIBEYE A-domain puncta densities in the OPL were determined using RIBEYE A-domain immunolabeled sections at higher magnifications that allow clear identification of single, immunolabeled puncta (Figures 6A–C,E), as described above for RIBEYE B-domain puncta density quantifications. Quantitative analyses revealed that the number of RIBEYE A-domain puncta in the OPL were indistinguishable between RBE^WT/WT^ and heterozygous RBE^WT/KI^ mice (Figure 6D). In contrast, RIBEYE A-domain puncta were completely absent from the OPL of RBE^KI/KI^ mice (Figure 6D). The results obtained with the rabbit polyclonal antibody against RIBEYE A-domain (Figures 6A–C) were confirmed by immunolabeling experiments with the monoclonal RIBEYE A-domain antibody 6F4 that also demonstrated the absence of RIBEYE A-domain puncta in the OPL (Figure 6E ).

These immunolabeling results, i.e., the absence of RIBEYE A-/RIBEYE B-immunolabeled puncta in the OPL of RBE^KI/KI^ mice, that we consistently obtained with anti-RIBEYE B-domain (Figures 2, 3) and anti-RIBEYE A-domain antibodies (6F4 and tau; Figures 4–6) indicate that no structures resembling synaptic ribbons are formed in photoreceptor synapses of RBE^KI/KI^ mice. To further test this conclusion, we performed ultrastructural analyses on rod photoreceptor synapses of the retinas from littermate RBE^WT/WT^, RBE^WT/KI^, and RBE^KI/KI^ mice by transmission electron microscopy (TEM; Figure 7).

TEM confirmed the absence of synaptic ribbons in photoreceptor synapses of RBE^KI/KI^ mice (Figures 7C1,C2), whereas synaptic ribbons were clearly present in RBE^WT/WT^ mice (Figures 7A1,A2) and heterozygous RBE^WT/KI^ (Figures 7B1,B2) (for quantification, see Figure 7D). Also, E-PTA-stained samples confirmed the absence of synaptic ribbons from the active zone of rod synapses in RBE^KI/KI^ mice (Figure 7C3). Synaptic ribbons were clearly present in E-PTA-stained rod photoreceptor synapses of RBE^WT/WT^ mice and heterozygous RBE^WT/KI^ mice (Figures 7A3,B3). From these experiments, we conclude that the RIBEYE B-domain is essential for the formation of synaptic ribbons in photoreceptor synapses.

As shown above, RIBEYE A- and B-domain immunosignals in the OPL were consistently weaker in heterozygous RBE^WT/KI^ mice in comparison to wild-type RBE^WT/WT^ mice (Figures 2, 4), whereas the ribbon density in the OPL was identical (Figures 3, 6). In order to identify possible reasons for the decreased RIBEYE immunosignals in the OPL of RBE^WT/KI^ mice in comparison to RBE^WT/WT^ mice, we measured the length of individual rod synaptic ribbons by 3D SR-SIM, as previously described (Dembla et al., 2020; Mukherjee et al., 2020; Kesharwani et al., 2021). For contour length measurements by 3D SR-SIM, rod synaptic ribbons were immunolabeled with either anti-RIBEYE B-domain (Figure 8) or anti-RIBEYE A-domain antibodies (Figure 9). By applying quantitative 3D SR-SIM analyses, we found that the contour length of synaptic ribbons was smaller in heterozygous RBE^WT/KI^ compared to RBE^WT/WT^ mice. This was consistently observed with both antibodies against RIBEYE (Figures 8, 9). This finding suggests that a decrease in the gene dosage of functional RIBEYE causes a decrease in the ribbon size, raising the exciting possibility that the ribbon size depends, among others, on the RIBEYE concentration.

In order to further characterize and confirm these light-microscopical alterations of synaptic ribbon size in photoreceptor synapses of RBE^WT/KI^ mice, we performed ultrastructural analyses by using TEM. We determined the height of synaptic ribbons in cross-sections of rod photoreceptor synapses of RBE^WT/WT^ mice and RBE^WT/KI^ mice. The height of cross-sectioned ribbons was measured from its anchorage site in the active zone to the free cytosolic end. For the determination of ribbon height, we applied the same criteria as previously defined (Kesharwani et al., 2021). Only rod photoreceptor synaptic ribbons were included that were anchored to a clearly visible active zone and were opposed by clearly visible postsynaptic triads. This procedure was applied to exclude those tangentially sectioned ribbons (sectioned parallel to the active zone) that were erroneously included in the analyses. We found that the ribbon height in rod photoreceptor synapses of heterozygous RBE^WT/KI^ mice was significantly lower (≈28%) than in RBE^WT/WT^ mice (Figures 10A,B; for quantification, Figure 10C).

The previous experiments consistently showed the absence of RIBEYE immunosignals in the OPL of RBE^KI/KI^ mice (Figures 2–6). As mentioned above, the OPL of the mouse retina contains mostly rod synapses, but also the less abundant cone synapses. In order to exclude the possibility that cone synapses might have escaped our attention in the immunolabeling experiments, we directly visualized cone terminals in the OPL by staining with fluorescent PNA lectin (Grabner et al., 2015) and analyzed with anti-RIBEYE 6F4 whether the cone terminals show RIBEYE immunosignals (Figures 11A1,A2). Despite the presence of PNA-positive cone terminals, no RIBEYE signals were observed in the OPL of RBE^KI/KI^ mice (Figure 11A2). The OPL of RBE^WT/WT^ mice served as a positive control in these experiments (Figure 11A1). Ribbons could be readily detected in the cone terminals of RBE^WT/WT^ mice (Figure 11A1) but not in the cone terminals of RBE^KI/KI^ mice (Figure 11A2) indicating the absence of synaptic ribbons also in cone synapses, similar to rod synapses.

To complement these analyses in the OPL, we analyzed synaptic ribbons in the inner plexiform layer (IPL) of the retinas from RBE^WT/WT^, RBE^WT/KI^, and RBE^KI/KI^ mice. For this purpose, we made use of the immunolabeling experiments that were shown in Figures 2, 4. In the IPL, 15 different types of retinal bipolar cells (Shekhar et al., 2016) form a morphologically and functionally heterogeneous population of synapses (Moser et al., 2020) that mostly contain synaptic ribbons at their active zone (Okawa et al., 2019). Synaptic ribbons are typically smaller in the IPL than in the OPL and exhibit different shapes, ranging from bar-shaped to ovoid (Moser et al., 2020). In agreement with the findings in the OPL, we observed a complete lack of both RIBEYE A-domain and RIBEYE B-domain immunosignals in the IPL of RBE^KI/KI^ mice in comparison to RBE^WT/KI^ and RBE^WT/WT^ mice (Figures 2, 4, 11B–E). We detected no RIBEYE fluorescence signals in the IPL of RBE^KI/KI^ mice (Figure 11) as expected. Electron microscopy confirmed the absence of synaptic ribbons in retinal bipolar cells, as shown by exemplary representative EM images of rod bipolar cells in Figures 11F–G. In RBE^WT/KI^ mice, RIBEYE fluorescence signals were again less intense than in the RBE^WT/WT^ mice, and RIBEYE puncta were also slightly, but highly significantly reduced (≈9% RIBEYE B puncta, ≈12% for RIBEYE A puncta) in comparison to homozygous RBE^WT/WT^ littermate control mice (for quantification, Figures 11B–E).

In order to determine whether deletion of the RIBEYE B-domain in RBE^KI^ mice has an impact on the expression levels of RIBEYE proteins, we performed Western blotting analyses on retinal lysates obtained from RBE^WT/WT^, RBE^WT/KI^, and RBE^KI/KI^ mice with the indicated primary antibodies using the Li-Cor system (Figure 12). As expected, we did not observe any RIBEYE band at ≈120 kDa with the anti-RIBEYE B-domain antibody in RBE^KI/KI^ mice (Figure 12B; for quantification, Figure 12F) because the RIBEYE B-domain is absent in RBE^KI/KI^ mice. In contrast, the RIBEYE band was clearly present in the lysates from littermate RBE^WT/WT^ and RBE^WT/KI^ mice (Figure 12B, arrowhead). With the anti-RIBEYE A-domain antibody, a RIBEYE A-domain-positive immunoblotting band was clearly present in retinal lysates from RBE^KI/KI^ mice, although the amount was less than in RBE^WT/WT^ mice and RBE^WT/KI^ mice (Figure 12C arrowhead; for quantification, Figure 12G). Antibodies against GFP were used to detect the expression of RIBEYE-GCaMP3 expression construct (Figure 12A, arrowhead). For normalization, the intensity of the respective bands obtained in the Li-Cor system was normalized to actin that served as a loading control. Importantly, the immunoblotting experiments revealed that the RIBEYE A-domain/GCaMP3 fusion protein was clearly produced in the RBE^KI/KI^ mice, but that the levels of the fusion protein were ≈31% of the wild-type RIBEYE levels (Figures 12C,G1,G2). These results suggest that the RIBEYE A-domain/GCaMP3 fusion protein may be unstable, possibly because it cannot be assembled into synaptic ribbons in the absence of the B-domain. The absence of synaptic ribbons in RBE^KI/KI^ mice is thus not due to a lack of RIBEYE protein synthesis but caused by the deletion of the B-domain.

Finally, we also analyzed synaptic ribbons in inner and outer hair cells (IHC and OHCs) in the organ of Corti of the inner ear. Synaptic ribbons were clearly visible in the IHCs and OHCs of RBE^WT/WT^ mice but completely absent in the IHCs and OHCs of RBE^KI/KI^ mice (Figure 13). This was consistently observed with both antibodies against RIBEYE B-domain (Figures 13A,B) as well as with antibodies against RIBEYE A-domain (Figures 13C,D).

Thus, synaptic ribbons were consistently absent in retinal synapses, both in the OPL and IPL, as well as in ribbon synapses in the IHCs and OHCs of the organ of Corti. These data show that the RIBEYE B-domain is essential for the assembly of synaptic ribbons.

## Discussion

RIBEYE is the defining protein component of synaptic ribbons that is essential for the formation of synaptic ribbons, as most directly shown with RIBEYE knockout mice in which deletion of RIBEYE leads to a complete absence of synaptic ribbons (Maxeiner et al., 2016; Becker et al., 2018; Jean et al., 2018). Although the fundamental role of RIBEYE in synaptic ribbons is thus well documented, the contribution of its individual protein domains and the mechanism of its action in ribbon assembly remains unclear. RIBEYE consists of a unique, proline-rich N-terminal RIBEYE A-domain and a C-terminal B-domain that is identical to CtBP2, a ubiquitously expressed transcriptional co-repressor. In support of the role of RIBEYE as a central building block of synaptic ribbons, multiple binding sites were identified in RIBEYE that mediate assembly RIBEYE multimers. In particular, several interaction sites were found in the RIBEYE A-domain that enable binding of the A-domain to other A-domains and to B-domains, which may be involved in ribbon assembly (Magupalli et al., 2008). Moreover, the RIBEYE B-domain/CtBP2 was shown to also interact with other B-domains and assemble into oligomeric structures (Kumar et al., 2002; Balasubramanian et al., 2003; Nardini et al., 2003; Madison et al., 2013; Bellesis et al., 2018; Jecrois et al., 2021). It seems likely that the RIBEYE A-domain constitutes the core assembly domain of the synaptic ribbon (Schmitz et al., 2000), but the role of the RIBEYE B-domain with its NAD(H) binding site in synaptic ribbons remains unknown. Indeed, it is even unclear whether the B-domain is required for synaptic ribbon assembly, or performs a different function associated with synaptic ribbons. For example, it is conceivable that the RIBEYE A-domain solely mediates the assembly of synaptic ribbons, and that the B-domain performs a peripheral function in tethering vesicles to the ribbon, an exciting possibility in view of the current lack of information on how synaptic ribbons organize continuous neurotransmitter exocytosis.

In the present study, we tested the basic role of the RIBEYE B-domain in mice. Using a genetic approach, we found that the RIBEYE B-domain is essential for the assembly of synaptic ribbons. Analysis of RIBEYE knockin (RBE^KI^) mice in which the B-domain of RIBEYE was replaced by an unrelated protein (the Ca^2+^-sensor GCaMP3) revealed a complete absence of synaptic ribbons even though the RIBEYE A-domain continues to be expressed as a GCaMP3 fusion protein. Thus, the RIBEYE A-domain is not sufficient for ribbon assembly, and the phenotype caused by the deletion of the B-domain is as severe as that of the complete RIBEYE deletion (Maxeiner et al., 2016). Moreover, our data confirm that RIBEYE functions universally in all synaptic ribbons as previously suggested (Schmitz et al., 2000; Maxeiner et al., 2016; Becker et al., 2018; Jean et al., 2018), since, in RBE^KI/KI^ mice, synaptic ribbons were completely absent not only in the retina but also in the inner and outer hair cells of the organ of Corti (Figure 13).

Interestingly, we show that one genomic copy of RIBEYE B-domain is largely sufficient to enable the assembly of synaptic ribbons, but that the ribbons in heterozygous RIBEYE^WT/KI^ mice are smaller. In photoreceptor synapses, the number of ribbons (ribbon density) were unchanged in the heterozygous RIBEYE^WT/KI^ mice but decreased in size in comparison to RIBEYE^WT/WT^ mice (as judged by quantitative 3D SR-SIM and EM measurements; Figures 8–10). This decrease in ribbon size in heterozygous RIBEYE^WT/KI^ could be explained by two alternative hypotheses. It is possible that the decreased expression of RIBEYE protein in the retina of RIBEYE^WT/KI^ mice in comparison to RIBEYE^WT/WT^ mice translates into a difference in ribbon size. This would imply that the ribbon size is determined by the RIBEYE concentration similar to a mass-action-law. Alternatively, it is possible that trace amounts of the RIBEYE A-domain/GCaMP3 fusion protein are incorporated into the ribbons, and that they hinder the full assembly of the ribbons. However, the fact that smaller synaptic ribbons were also reported in heterozygous RIBEYE knockout mice (RBE^WT/KO^) compared to RIBEYE WT mice (RBE^WT/WT^) (Jean et al., 2018) strongly supports the first hypothesis. In further support of this hypothesis, moreover, transgenic overexpression of RIBEYE protein in zebrafish results in particularly large, oversized synaptic ribbons (Sheets et al., 2011, 2017). In the IPL of the inner retina, the fluorescence intensity of RIBEYE immunosignals was decreased in RIBEYE^WT/KI^ mice in comparison to RIBEYE^WT/WT^ mice. In the IPL, the number of synaptic ribbons/per IPL area was smaller in heterozygous RIBEYE^WT/KI^ mice in comparison to RIBEYE^WT/WT^ mice. This is probably based on the fact that synaptic ribbons in bipolar terminals in the IPL are typically smaller in size than in rod photoreceptor terminals (Moser et al., 2020). A further reduction in size in heterozygous RIBEYE^WT/KI^ mice thus can more easily result in their complete disappearance in bipolar cell synapses than in rod photoreceptor synapses in which synaptic ribbons are bigger.

In summary, our results demonstrate that the RIBEYE B-domain is essential for the assembly of synaptic ribbons and that the RIBEYE A-domain alone cannot build synaptic ribbons in the synaptic terminals of the retina and in sensory hair cells of the inner ear. How the RIBEYE B-domain promotes ribbon assembly needs to be further analyzed, but it seems likely that it acts as more than a structural component. Recent biochemical and structural analyses on CtBP1 and CtBP2, that is largely identical to the RIBEYE B-domain, can give important hints (Nardini et al., 2009; Madison et al., 2013; Bellesis et al., 2018; Jecrois et al., 2021). These data showed that CtBP2 forms tetrameric complexes via dimeric intermediates (Madison et al., 2013; Nichols et al., 2021). The assembly of these complexes is enhanced by NAD(H) (Madison et al., 2013; Jecrois et al., 2021; Nichols et al., 2021). Interestingly, NAD(H) has been shown to influence the size of synaptic ribbons in sensory hair cells (Wong et al., 2019; Okur et al., 2020). The size of auditory hair cell ribbon synapses also strongly increases with aging in C57BL/6J mice (Peineau et al., 2021). Based on the CtBP1/CtBP2 structural data (Balasubramanian et al., 2003; Madison et al., 2013; Jecrois et al., 2021; Nichols et al., 2021), the RIBEYE B-domain can be expected to also assemble into tetrameric complexes that are controlled by NAD. These oligomeric RIBEYE B-complexes could link different RIBEYE units to each other during ribbon assembly and/or mediate stability to the synaptic ribbon. The functional properties of ribbon synapses in RBE^KI/KI^ mice and the physiological consequences of RIBEYE B-domain deletion in the RBE^KI/KI^ mice for vision and hearing need to be analyzed by future analyses.

## Data Availability Statement

The original contributions presented in the study are included in the article, further inquiries can be directed to the corresponding author/s.

## Ethics Statement

The animal study was reviewed and approved by Landesamt für Verbraucherschutz; Geschäftsbereich 3; 66115 Saarbrücken, Germany; GB 3-2.4.1.1-K110/180-07.

## Author Contributions

SS performed all experiments and analyses shown in Figures 1–13 and wrote the article together with FS and TS. KS performed embeddings and provided help with experiments and statistical procedures. RK introduced SS to confocal microscopy and provided help with analyses. MJ generated the peptide arrays and provided important advice. SM generated knockin and knockout mice and provided help with analyses. TS designed knockin strategy, organized KI and KO mouse generation and provided essential support. FS designed and supervised the study and wrote the article together with SS and TS. All authors contributed to the article and approved the submitted version.

## Conflict of Interest

The authors declare that the research was conducted in the absence of any commercial or financial relationships that could be construed as a potential conflict of interest.

## Publisher’s Note

All claims expressed in this article are solely those of the authors and do not necessarily represent those of their affiliated organizations, or those of the publisher, the editors and the reviewers. Any product that may be evaluated in this article, or claim that may be made by its manufacturer, is not guaranteed or endorsed by the publisher.

## Footnotes

^1^ https://astatsa.com/WilcoxonTest/
